# Evaluation of Alignment Algorithms for Discovery and Identification of Pathogens Using RNA-Seq

**DOI:** 10.1371/journal.pone.0076935

**Published:** 2013-10-30

**Authors:** Ivan Borozan, Stuart N. Watt, Vincent Ferretti

**Affiliations:** Informatics and Bio-computing, Ontario Institute for Cancer Research, Toronto, Ontario, Canada; Georgia Institute of Technology, United States of America

## Abstract

Next-generation sequencing technologies provide an unparallelled opportunity for the characterization and discovery of known and novel viruses. Because viruses are known to have the highest mutation rates when compared to eukaryotic and bacterial organisms, we assess the extent to which eleven well-known alignment algorithms (BLAST, BLAT, BWA, BWA-SW, BWA-MEM, BFAST, Bowtie2, Novoalign, GSNAP, SHRiMP2 and STAR) can be used for characterizing mutated and non-mutated viral sequences - including those that exhibit RNA splicing - in transcriptome samples. To evaluate aligners objectively we developed a realistic RNA-Seq simulation and evaluation framework (RiSER) and propose a new combined score to rank aligners for viral characterization in terms of their precision, sensitivity and alignment accuracy. We used RiSER to simulate both human and viral read sequences and suggest the best set of aligners for viral sequence characterization in human transcriptome samples. Our results show that significant and substantial differences exist between aligners and that a digital-subtraction-based viral identification framework can and should use different aligners for different parts of the process. We determine the extent to which mutated viral sequences can be effectively characterized and show that more sensitive aligners such as BLAST, BFAST, SHRiMP2, BWA-SW and GSNAP can accurately characterize substantially divergent viral sequences with up to 15% overall sequence mutation rate. We believe that the results presented here will be useful to researchers choosing aligners for viral sequence characterization using next-generation sequencing data.

## Introduction

Emerging and re-emerging infectious diseases in the past three decades have created a significant cause of concern worldwide and exerted a significant burden on public health. In the past decade alone, we have seen epidemics of virus variants such as the avian influenza H5N1 and the swine flu H1N1 that still pose a significant threat to the public health [1]. Furthermore some infectious agents such as viruses have been found to be etiological agents of human cancer, causing 15% to 20% of all human tumors worldwide [2]. Despite significant progress in the fight against infectious diseases there is clearly a pressing need for fast and accurate methods in the discovery and identification of viral etiological agents.

Traditional methods for virus identification lack the sensitivity required to detect viruses that are present in low abundance, and are biased towards viruses with known sequences (see [3]). These methods are thus not suitable for the detection of viruses that are entirely novel, or that differ by a significant number of mutations in their key primary regions. In contrast, Next-generation sequencing (NGS) technologies provide an unequaled opportunity for the identification of known and novel viruses, in an unbiased way, with high sensitivity, and have recently been successfully applied in the discovery of novel viral agents (see [4]–[6]). Viral sequences can be sequenced from either total DNA (for DNA viruses) or RNA isolated from host organisms (e.g. human, insects, etc.) and bioinformatics analysis plays a crucial role in the processing, analysis and characterization of such sequences. In principle either whole genome or transcriptome sequencing (RNA-Seq) can be used for viral identification, however whole transcriptome sequencing can be performed at a fraction of the cost of whole genome sequencing and is particularly suitable for identifying viruses that are actively expressed in the host making them more likely to be involved in the etiology of disease.

A number of recently published methods for pathogen identification in humans [7]–[10] that use NGS data rely on a computational approach known as digital subtraction [11]. Digital subtraction consists of subtracting 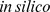 known human sequences from human transcriptome or genome (DNA) sequence data, leaving candidate non-human sequences to be aligned against known pathogen reference sequences. In this approach reads that do not align to human and pathogen reference sequences can be assembled 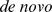 into contigs, large contigs might be indicative of new and as yet undetected organisms.

All digital subtraction is based on the use of alignment algorithms (e.g. PATHSEQ [7] uses MAQ[12] and BLAST [13], RINS [8] uses Bowtie [14] and BLAT [15], CaPSID [9] can use any aligner - as long as it satisfies certain criteria) and choosing the most suitable one plays a key role in viral identification and discovery. Choosing the right aligner, however, needs to be based on an objective evaluation and comparison of different aligner tools.

Furthermore, while bacterial and eukaryotic organisms have mutation rates that are between 10^−7^ to 10^−10^ mutations per base per generation [16], [17] the observed mutation rates for both RNA and DNA viruses are much higher (for DNA viruses mutation rates are between 10^−6^ to 10^−8^ mutations per base per generation, and for RNA viruses between 10^−3^ to 10^−5^ per base per generation [16]). It is therefore important to evaluate the ability of various aligners to correctly characterize more highly mutated viral transcripts, some of which can even exhibit RNA splicing.

The aims of this article are thus threefold 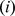 evaluate and understand the extent to which alignment algorithms can be used for characterizing mutated and non-mutated viral sequences, including those that exhibit splicing 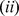 to evaluate and compare the performance of alignment algorithms in terms of their accuracy, sensitivity, precision and runtime 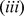 identify the best overall aligner (or set of aligners) for digital subtraction.

To address these three objectives we developed a realistic RNA-Seq simulation and evaluation framework (RiSER). RiSER uses the ART [18] NGS read simulator, which emulates a sequencing process using technology-specific error models for indels, SNPs and base quality values, but augments it with the capability to simulate RNA-Seq data. To the best of our knowledge the only other realistic RNA-Seq simulator published to date is BEER [19]. BEER, however, cannot be used for data simulation of reference genomes other than human.

Although recently published benchmarking studies [19], [20] did assess and compare the performance of different aligners, none of these approaches were suitable (or could be easily modified) for evaluating aligners' performance in identifying mutated and non-mutated viral sequences in transcriptome samples. For example SEAL [20] is designed to evaluate alignment tools using simulated genomic data only, while BEER is designed to evaluate alignment tools using transcriptome data that can only be simulated from the human reference genome. In this manuscript we describe the RiSER framework, and use it to evaluate and rank the performance of eleven alignment algorithms (BLAST [13], BLAT [15], BWA [21], BWA-SW [22], BWA-MEM [23], BFAST [24], SHRiMP2 [25], Bowtie2 [14], GSNAP [26], Novoalign (Novocraft) and STAR [27]) using a set of metrics, which we have specifically designed to address the above three objectives.

## Results

Viral genomes are generally less complex than eukaryotic ones, they have limited or no splicing and should present less of a challenge for aligner algorithms to align short reads to their reference sequences. With this in mind we selected eleven different alignment algorithms to evaluate (see Materials and Methods section for criteria used) using RiSER, five of which are capable of aligning reads across splice junctions (BLAST, BLAT, BFAST, GSNAP and STAR), and six that are not (BWA, BWA-SW, BWA-MEM, SHRiMP2, Bowtie2 and Novoalign), using the four different viral sequences shown in Table 1. To evaluate the ability of aligners to align reads across splice junctions in the context of viral sequences, we chose two viral genomes (HIV-1 and Human papillomavirus-18, see Tables 1 and 2) which have been shown to generate distinctly complex patterns of spliced RNA to encode some of their essential regulatory proteins [28], [29].

**Table 1:** Information about four different viruses used in this study.

Group	Virus name	Accession	Genome size (bp)	Splicing
dsDNA	Human papillomavirus-18 (HPV18)	NC_001357.1	7857	Yes
dsDNA	Human herpesvirus 1 (HSV1)	NC_001806.1	152261	No
ssRNA(-)	Influenza A virus (A/avian/Hong Kong/0719/2007(H5N1)) segment 4	GU050317.1	1751	No
ssRNA-RT	HIV-1 vector pNL4-3 (HIV-1)	AF324493.2	14825	Yes

**Table 2:** Summary of data generated for each of the four viral genomes.

Accession	Virus name	Genome size (bp)	Number of transcripts	Number of spliced transcripts	Number of reads generated	Number of reads crossing splice junctions
NC_001357.1	HPV18	7857	8	15	3480	219±17
NC_001806.1	HSV1	152261	77	0	11960	0
GU050317.1	H5N1 seg-4	1751	1	0	170	0
AF324493.2	HIV1	14825	10	43	5760	922±15

Shows the summary of data generated for each viral genome (see also Table 1) using RiSER's RNA-Seq simulation approach. For each virus we show the genome size, the total number of transcripts (spliced and un-spliced), the total number of reads generated (for a sequencing depth of 10 fold), and the average number of reads that cross splice junctions (the average value is calculated across 10 runs).

To evaluate aligners in the context of the digital subtraction we designed a realistic RNA-Seq simulated benchmark dataset that is representative of typical datasets for pathogen identification in humans using whole transcriptome sequencing (or RNA-Seq). In this respect, our dataset includes three distinct sets of simulated NGS reads: 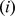 reads mapping to the human host reference genome (hg19), 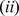 reads mapping to the human host genomic regions not represented in the hg19 reference genome and 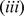 non-human reads mapping to viruses that the experiment is designed to characterize - including reads mapping to a host retrovirus not in the human host reference genome (i.e., the HIV-1 virus) as shown in Tables 1 and 2.

### Evaluation of aligners' performances using RNA-Seq reads simulated from non-mutated viral genomes

To simulate reads mapping to viruses that the experiment is designed to characterize (i.e. part 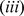 of our benchmark dataset as explained in the previous paragraph) we used our RNA-Seq simulation approach, as described in the Materials and Methods section, to generate Illumina-type realistic sequencing datasets (single-end reads, read length = 100 bp, total sequence coverage = 10 fold, replicates = 10) from each viral genome shown in Table 1.

Table 2 shows the summary of data generated for each viral genome using RiSER's RNA-Seq simulation approach. As expected, viruses with the highest number of spliced transcripts also have the largest proportion of simulated reads that cross splice junction regions. Because most viruses exhibit limited or no splicing, it is expected that the majority of reads for any given sequenced virus will not cross a splice junction.

**Figure 1:**
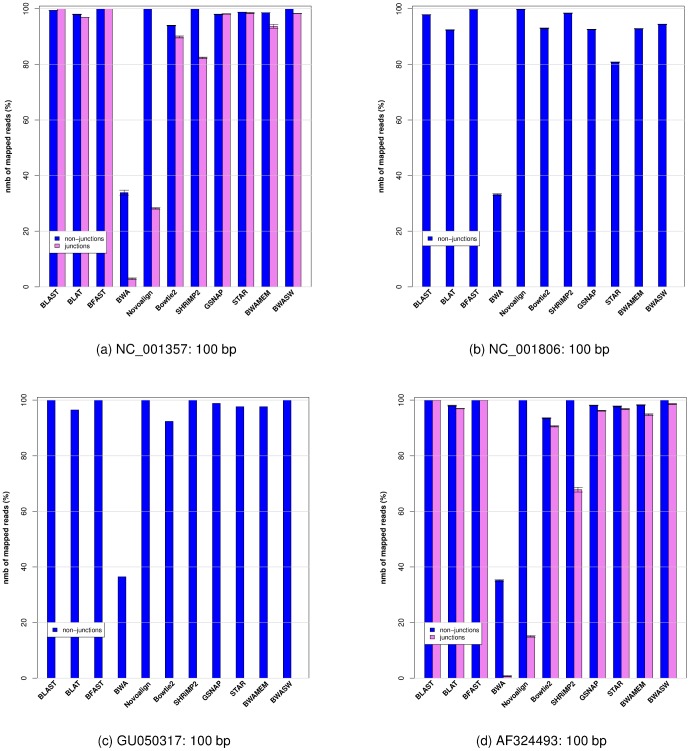
Histogram plot of the percentage of reads aligned (averaged over 10 runs) for each viral genome. Histogram plot of the percentage of reads aligned (averaged over 10 runs) for each viral genome shown in Table 2 and each aligner. Reads crossing splice junction regions are shown in pink, reads not crossing splice junction regions are shown in blue.

In Figure 1 we show for each individual virus the histogram of the percentage of aligned reads averaged over 10 runs for each aligner, each aligner was run with the moderate sensitivity parameter value settings (for the detailed description of parameter value settings used for each aligner see section 4 in the Materials S1).

We first observe that aligners performed consistently across all four viral genomes for both sets of reads (i.e., for reads that are crossing a splice junction and for those that are not). Second most aligners (with the exception of BWA) align reads that do not cross a splice junction with a high success rate, although we observed some small variation in performance between aligners.

The results shown in Figure 1 separate into roughly two groups, aligners that have the ability to align the simulated reads from our benchmark dataset across splice junctions (Group 1: BLAST, BLAT, BFAST, GSNAP, STAR, BWA-SW and BWA-MEM), and aligners that have limited or no ability to align simulated reads across splice junctions (Group 2: BWA and Novoalign). Bowtie2 and SHRiMP2 lie somewhere in between these two groups, even though these two algorithms together with BWA-SW and BWA-MEM were not designed to align reads across splice-junctions.

**Figure 2:**
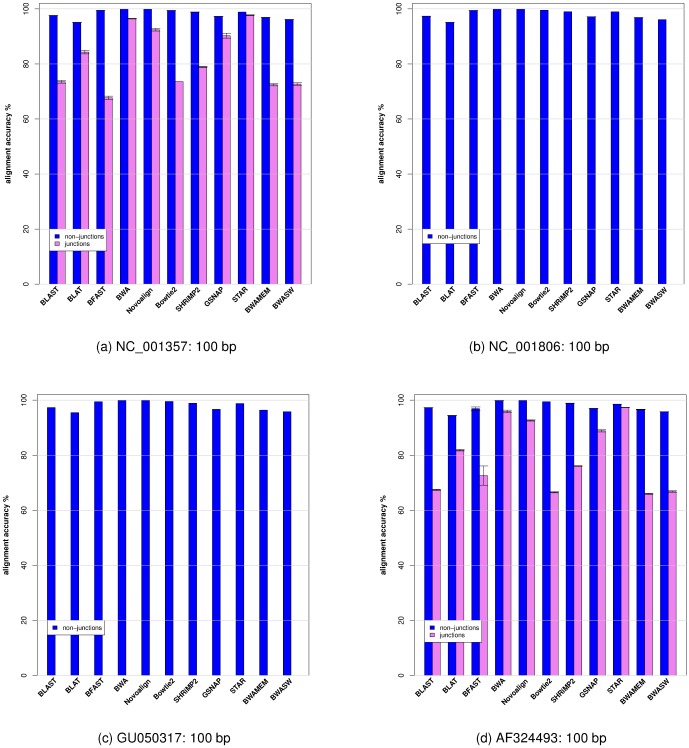
Histogram plot of the average alignment accuracy (averaged over 10 runs) for each viral genome. Histogram plot of the average alignment accuracy averaged over 10 runs for each viral genome shown in Table 1 and each aligner. Reads crossing splice junction regions are shown in pink, reads not crossing splice junction regions are shown in blue).

In Figure 2 we show the alignment accuracy (see the Materials and Methods section for how alignment accuracy is calculated) averaged over 10 runs. We note that overall aligners (including the aligners in Group 1) achieved higher alignment accuracy for reads that do not cross a splice junction than for those that do.

In order to measure the overall performance of individual aligners we introduce two scores: S1 and S2. We define the S1 score as a measure of aligner's performance expressed as the geometric mean between the percentage of reads aligned outside splice junction regions (i.e., 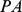 see Materials and Methods section) and their average alignment accuracy (i.e., 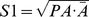), and S2 as the geometric mean between the percentage of reads aligned across splice junction regions (i.e., 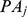 see Materials and Methods section) and their alignment accuracy (i.e., 
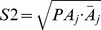
). The choice of the geometric mean is based on the observation that both the percentage of reads aligned and the accuracy of the read's alignment are normalized ratios (i.e., the percentage of reads aligned is normalized to the total number of reads simulated from a given reference sequence, and the alignment accuracy is normalized to the total number of bases contained by the read; see Materials and Methods section). Use of the geometric mean also prevents numeric ranges of either of these two variables (i.e., the percentage of the reads aligned and alignment accuracy) from dominating the weighting when calculating the S1 and S2 scores. In order to rank aligners we aggregate the results shown in Figures 1 and 2 across the four viral genomes, and present them in Table 3.

**Table 3:** Summary of S1 and S2 scores for viral reference sequences.

Aligner	*PA* (percentage of aligned reads (non-junctions)) (%)	*PA_j_* (percentage of aligned reads (junctions)) (%)	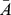 non-junctions (%)	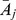 (junctions) (%)	S1	S2
Novoalign	99.96±0.04	21.62±0.89	99.92±0.01	92.52±1.18	99.94±0.02	44.73±0.96
BFAST	99.94±0.03	99.97±0.08	98.82±0.95	70.16±7.92	99.38±0.48	83.75±4.73
SHRiMP2	99.61±0.12	75.07±2.00	98.90±0.06	77.48±0.51	99.26±0.07	76.27±1.04
BLAST	98.93±0.09	99.53±0.18	97.41±0.06	70.26±1.26	98.17±0.06	83.63±0.75
BWASW	98.6±0.13	98.45±0.39	95.96±0.04	69.76±1.17	97.27±0.07	82.87±0.71
GSNAP	96.87±0.29	97.17±0.37	97.03±0.10	89.55±2.18	96.95±0.15	93.28±1.15
BWAMEM	96.83±0.20	94.24±1.73	96.7±0.15	69.2±1.00	96.77±0.13	80.76±0.94
Bowtie2	93.21±0.38	90.23±0.96	99.49±0.03	70.07±0.50	96.30±0.20	79.51±0.51
STAR	93.74±0.33	97.63±0.65	98.79±0.04	97.56±0.33	96.23±0.17	97.59±0.36
BLAT	96.22±0.34	96.95±0.18	95.01±0.15	83.09±1.64	95.61±0.19	89.75±0.89
BWA	34.67±1.58	1.82±0.77	99.94±0.06	96.18±1.10	58.86±1.34	13.23±2.80

Shows the summary of alignment results aggregated over four viral genomes for non-mutated viral reference sequences and sorted by S1 score. The average alignment accuracy for reads crossing splice junctions (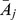) and those not crossing splice junctions (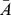) is defined in the Materials and Methods section.

Ranked by the S1 score (see Table 3) the two best aligners are Novoalign and BFAST followed closely in decreasing order by SHRiMP2, BLAST, BWA-SW, GSNAP, BWA-MEM, Bowtie2, STAR and BLAT. BWA has a significantly lower S1 score. Though BWA is highly accurate its S1 score is low because it aligns only a small fraction of reads, which can be explained by the high stringency with which BWA is designed to align reads (short queries up to 200 bp with a low error rate, 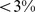). If the aligners are ranked by S2 the four best aligners are, as expected, STAR, GSNAP, BLAT, BFAST and BLAST. We note that among algorithms that are not designed to align reads across splice junction 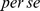, Bowtie2, BWA-SW and BWA-MEM align a good proportion of reads (90.23%, 98.45% and 94.24% respectively) with similar alignment accuracy to that of BFAST. The performance of these three aligners to align spliced reads can be explained by their ability to perform local alignments and align reads with an unrestricted number of gaps and gap lengths.

To investigate the extent to which the ranking presented in Table 3 is stable against variation in parameter value settings, we align the same set of reads using two additional parameter value settings: the default and high sensitivity (for the detailed description of parameter value settings used for each aligner see section 4 in the Materials S1). If we rank aligners by the S1 score we observe no significant change in the ranking when using either the default (see Table S1 in Materials S1) or high sensitivity (see Table S10 in Materials S1) parameter value settings, except for GSNAP which performs best when using moderate and high sensitivity parameter value settings. If ranking aligners by the S2 score we find that STAR has the most consistent performance, and that together with GSNAP (only for moderate and high sensitivity parameter value settings) these two algorithms show the best performance when aligning reads across splice junctions.

### Evaluation of aligners' performances using RNA-Seq reads simulated from mutated viral genomes

Note that for the purpose of this part of the evaluation we did not take splicing into account. As in the previous section we first run aligners with moderate sensitivity parameter value settings (for the detailed description of parameter value settings used for each aligner see section 4 in the Materials S1), then we investigate the ranking with two additional parameter value settings (default and high sensitivity).

**Figure 3:**
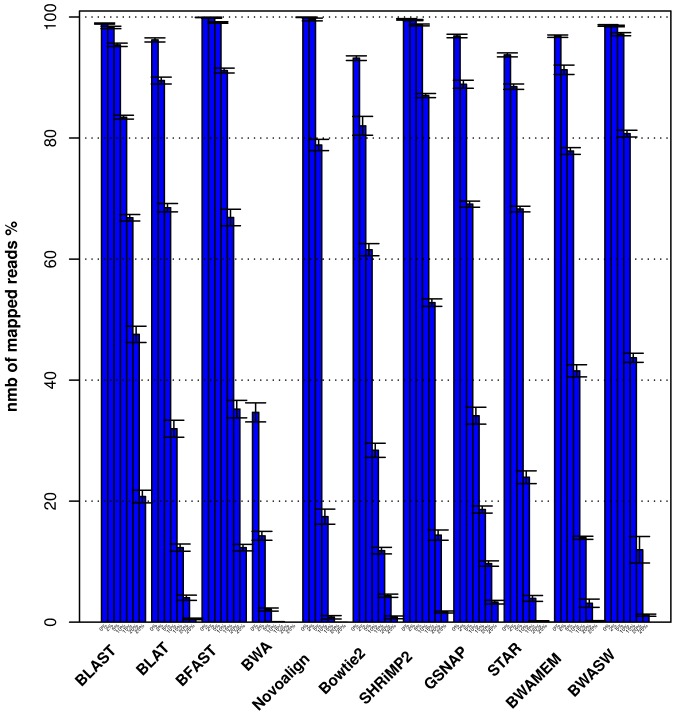
Histogram plot of the number of aligned reads as a function of the viral mutation rate. Histogram plot of the number of aligned reads as a function of the mutation rate for each aligner averaged over the four viral genomes (see Table 1).

**Figure 4:**
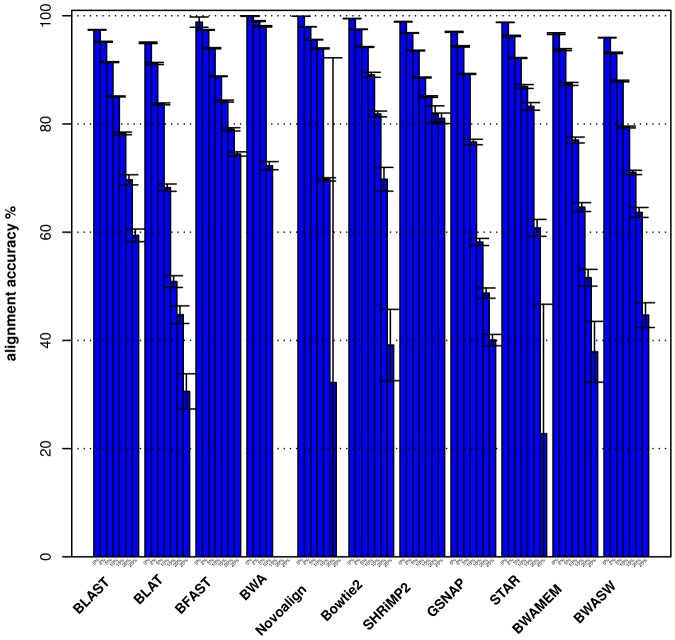
Histogram plot of the average accuracy as a function of the viral mutation rate. Histogram plot of the average accuracy as a function of the mutation rate for each aligner averaged over the four viral genomes (see Table 1).

As shown in Figure 3, the number of aligned reads correlates, as expected, negatively with the mutation rate. While BWA and Novoalign have the lowest percentage of reads aligned for mutation rates 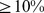, BLAST, BFAST and SHRiMP2 align consistently on average the greatest proportion of reads at each mutation rate across all four viral genomes. As expected for all the aligners we also observe a drop in the accuracy with which reads are aligned as the mutation rate increases (see Figure 4), and find that aligners such as GSNAP, BLAT, STAR, BWA-SW and BWA-MEM show a much steeper drop in accuracy than BFAST, SHRiMP2 and BLAST as the mutation rate increases. The same conclusions can be drawn from results obtained by running the aligners using default and high sensitivity parameter value settings, as shown in Figures S3, S4, S5, S6 in Materials S1.

In order to rank the performance of individual aligners in terms of their precision (see Table S7 in Materials S1), sensitivity (see Table S8 in Materials S1), S1 and S2 scores we use a measure 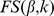 which combines these four metrics into a single score as defined below
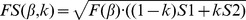
(1)where 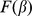 [30] is the harmonic mean of precision and recall (or sensitivity) and 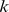 is the parameter (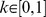) determined by the ratio of the number of reads crossing splice junction regions to those that do not – for viruses that exhibit RNA splicing. For the purpose of viral identification we set the 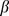 parameter in eq.(1) to one, in this way the 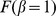 (or 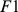) score measures the effectiveness with which an aligner identifies the correct viral reference sequence when the same weight is given to both precision and recall. The 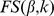 in eq.(1) is thus defined as a geometric mean between the 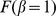 score and the weighted sum of the S1 and S2 scores. The 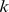 parameter in eq.(1) insures that the proportionate weight is given to the S1 and S2 scores based on the estimated amount of splicing. Thus for viruses that exhibit limited or no splicing the S1 score dominates over that of S2, and proportionally more weight is given to the S2 score as the amount of splicing increases. The 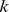 parameter in eq.(1) can be estimated for a single viral sequence or a population of viral sequences by taking the arithmetic mean of their individual 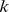 values.

Table 4 shows the values for 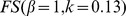 for each aligner as a function of the viral mutation rate. The value of the 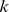 parameter for the data in Table 4 was estimated from the data presented in Table 2 using viruses that exhibit RNA splicing and by taking the arithmetic mean of their individual 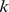 values.

**Table 4:** Summary of values for the 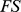 measure.

Aligner	Mutation (0%)	Mutation (2%)	Mutation (5%)	Mutation (10%)	Mutation (15%)	Mutation (20%)	Mutation (25%)
BLAST	89.72	90.15	90.46	87.36	78.96	62.48	34.56
BFAST	89.87	90.14	91	87.79	73.78	50.53	25.64
SHRiMP2	94.85	94.69	94.03	87.56	66.25	29.67	6.99
BWASW	96.76	94.33	89.06	67.43	39.43	13.38	2.9
GSNAP	97.75	93.14	80.96	52.74	33.06	20.16	8.92
BWAMEM	96	92.42	83.63	56.44	27.58	8.48	1.73
BLAT	96.81	92.34	79.22	49.05	24.3	9.93	3.03
Bowtie2	93.45	87.36	74.49	45.59	24.50	11.44	2.51
STAR	97.83	94.83	82.62	44.05	13.13	0.96	0
Novoalign	96.27	95.19	83.85	33.17	3.70	0.08	0
BWA	51.78	28.15	6.69	0.38	0	0	0

Shows values of the 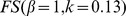 measure averaged over four viral genomes (see Table 2) as a function of viral mutation rates, sorted according to the average 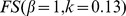 values for mutation rates 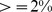.

In Table 4, we observe a consistent drop in the 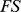 values across all aligners as the viral mutation rate increases with BLAST and BFAST showing the best performance when taking the average 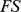 values for viral mutation rates 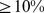. Because precision (see Table S7 in Materials S1) for the top five aligners (BLAST, BFAST, SHRiMP2, BWA-SW and GSNAP) remains high and approximately independent of the mutation rate, the drop in values of the 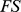 measure is due to a drop in recall (see Table S8 in Materials S1) caused by a drop in the number of aligned reads at higher mutation rates. At the low end of the mutation spectrum (i.e., mutation rate 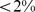), the top five aligners with highest scores are STAR, GSNAP, BLAT, BWA-SW and Novoalign, while BWA achieves a lower score than any other aligner considered in this study. As the parameter value settings are changed from moderate sensitivity to high (see Table S11 in Materials S1), the ranking of aligners for viral mutation rates 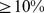 changes very little (BLAST and BFAST having the best scores) with the exception of BLAT which has higher 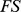 values due to an increase in sensitivity as shown in Table S14 in Materials S1. With default parameter settings the most significant drop in terms of 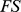 values is observed for GSNAP for both the high and low viral mutation rates (see Table S2 in Materials S1). In summary the aligners that benefit the most in terms of 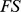 values from more sensitive parameter value settings are BLAST, BLAT and GSNAP.

Table 5 shows the total genome coverage obtained for each aligner as a function of the viral mutation rate. We define the genome coverage as the number of genome nucleotides represented in aligned reads normalized by the genome length. Note that the results in Table 5 are shown as ranges of observed genome coverage in terms of their minimum and maximum values (i.e., min, max) of the four viral genomes presented in Table 2.

**Table 5:** Genome Coverage.

Aligner	Mutation (0%)	Mutation (2%)	Mutation (5%)	Mutation (10%)	Mutation (15%)	Mutation (20%)	Mutation (25%)
BLAST	97–100	97–100	96–100	97–100	95–99	90–95	66–73
BFAST	97–100	97–100	96–100	97–100	94–98	76–85	49–55
SHRiMP2	97–100	97–100	96–100	97–100	87–94	47–57	6–14
BWASW	97–100	97–100	96–100	96–98	72–78	31–42	0–9
GSNAP	97–100	97–100	93–100	82–84	46–57	24–39	8–15
Bowtie2	97–100	96–100	94–99	77–84	42–51	13–21	0–6
BWAMEM	97–100	97–100	94–100	78–83	31–42	7–11	0–3
BLAT	97–100	97–100	93–99	71–74	23–34	7–12	0–3
STAR	97–100	97–100	96–100	69–72	19–28	0–4	0–0
Novoalign	97–100	97–100	92–99	45–54	0–10	0–1	0–0
BWA	96–97	65–69	7–20	0–1	0–0	0–0	0–0

The summary of values of genomes' coverage in terms of their minimum and maximum values (i.e., min-max) obtained by each individual aligner, averaged over four viral genomes (see Table 2) as a function of viral mutation rates and sorted according to the average coverage for mutation rates 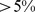.

Using moderate sensitivity parameter value settings the results in Tables 4 and 5 indicate that BLAST, BFAST, SHRiMP2, BWA-SW and GSNAP can be used to characterize highly mutated viral sequences (for mutation rates 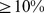 and 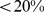) with high precision (see Table S7 in Materials S1) and high coverage (see Table 5) if sequenced at the depth of at least 10 fold. Of these five aligners we expect BLAST and BFAST to achieve the best performance when characterizing viral sequences with mutation rates 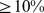. For high sensitivity parameter value settings the results shown in Table S11 and Table S12 in Materials S1 indicate that BLAST, BFAST, GSNAP, BWA-SW and BLAT show the best performance when characterizing highly mutated viral sequences (for mutation rates 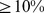 and 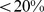) in terms of the 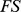 values, genome coverage and high precision (for precision see Table S13 in Materials S1).

In summary the best performing aligner is BLAST with high sensitivity parameter value settings, a close second best is BFAST with moderate sensitivity parameter value settings, the third best is SHRiMP2 with moderate sensitivity parameter value settings, the fourth is BWA-SW with high sensitivity parameter value settings, fifth is GSNAP with high sensitivity parameter value settings and sixth is BLAT with high sensitivity parameter value settings.

Furthermore, we found that using more conservative aligners to characterize increasingly divergent viral sequences results in a marked drop in their sequence coverage due to a high attrition rate of aligned reads, in addition to lowering the detection rate and providing more uncertain characterization.

### Evaluation of aligners' performances using RNA-Seq reads simulated from the human reference

In order to determine the best set of aligners for digital subtraction we also evaluated the ability of each individual aligner to subtract human sequences from the host human transcriptome sequence data. To simulate reads mapping to the human host reference genome (hg19) (i.e. part 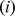 of our benchmarking dataset) we used the RiSER framework to simulate a single dataset with 20 million short reads (single-end reads, read length = 100 bp, total sequence coverage = 10 fold, replicates = 1) from the human (hg19) reference genome in the FASTA format. To simulate reads that cross splice junctions we generated the transcript information file from the Known Genes dataset [31] accessed from the UCSC website (http://hgdownload.cse.ucsc.edu/goldenPath/hg19/database/knownGene.txt.gz).

Because the evaluation process is computationally intensive we chose to evaluate aligners using a smaller set containing 2 million short reads simulated from the first human chromosome (chr1). For this section all aligners were evaluated using moderate sensitivity parameter value settings (for the detailed description of parameter value settings used for each aligner see section 5 in the Materials S1). A summary of the simulated reads for this part of the evaluation is shown in Table 6. Furthermore, we omitted the evaluation of the BLAST algorithm since it is not a commonly used aligner when aligning a very large number of NGS short reads against a single human reference genome[12], [32].

**Table 6:** Summary table for reads simulated from the human reference genome.

RefSeq accession	Chromosome name	Genome size (bp)	Number of transcripts	Number of reads generated	Number of reads crossing splice junctions
NC_000001	chr1	249250621	7536	1995040	599844

Table showing the summary of reads simulated using the RiSER€s framework and the first human chromosome (chr1) as the reference.

Table 7 shows the results of the evaluation of the ten aligners using simulated human (chr1) transcriptome data. GSNAP has the highest S2 score, indicating its ability to correctly align the majority of simulated RNA-Seq reads back to the human reference sequence in the absence of information about splice junctions. The other two algorithms with high S2 scores are BLAT and BFAST. However if the information about splice junctions is available, then aligners with the highest S1 scores and best performances are BFAST, SHRiMP2, Novoalign, STAR and GSNAP in descending order.

**Table 7:** Summary of S1 and S2 scores for the human reference genome.

Aligner	*PA* (percentage of aligned reads (non-junctions)) (%)	*PA_j_* (percentage of aligned reads (junctions)) (%)	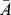 non-junctions (%)	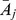 (junctions)	S1	S2
GSNAP	97.08	95.64	95.85	86.91	96.46	91.17
BLAT	82.35	84.58	94.77	83.92	88.35	84.25
BFAST	99.68	98.11	99.27	63.76	99.47	79.09
BWAMEM	97.19	93.00	95.70	66.78	96.44	78.80
Bowtie2	92.62	87.86	97.93	68.72	95.24	77.70
SHRiMP2	99.66	75.97	98.62	76.09	99.14	76.03
BWASW	93.96	83.50	94.87	68.09	94.42	75.40
STAR	97.85	54.68	97.74	88.73	97.80	69.65
Novoalign	99.13	37.43	98.79	86.06	98.96	56.75
BWA	35.01	1.37	98.81	93.41	58.82	11.30

The summary of alignment results for the human reference sequence (chr1) sorted by S2. The average alignment accuracy for reads crossing splice junctions (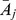) and those not crossing splice junctions (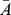) is defined in the Materials and Methods section.

Table 8 shows the values for precision, sensitivity and the 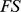 measure for each aligner using the simulated human transcriptome dataset. While for the purpose of viral characterization we assign the same weights to both precision and recall (see eq.(1)), for the simulated human transcriptome data we should score preferentially those aligners that have higher alignment rates (i.e., when aligning human derived reads back to the human reference sequence). To do this we set 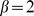 in eq.(1) to give twice as much emphasis on recall than precision. We also estimated the value for the 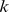 parameter (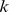 = 0.43) for the 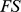 measure using data from Table 6. To calculate the precision and sensitivity, all simulated reads from the human reference genome were aligned against a database of 4195 known viral reference sequences, as described in the Materials and Methods section for simulated viral sequences. While all aligners in Table 8 have high precision, Novoalign and BWA show a drop in sensitivity due to their inability to align reads across splice junctions. This drop in sensitivity for Novoalign and SHRiMP2 (and to a much lesser extent, for all the other aligners) is reduced once the splice junction library is provided, as shown in Table 8.

**Table 8:** Precision, sensitivity and the 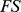 score for reads simulated from the human reference sequence.

Aligner	Precision (%)	Sensitivity (recall) (%)	Sensitivity with splice junction library (%)	FS(*β* = 2,k = 0.43) (without splice junction library)	FS(*β* = 2, k = 0) (with splice junction library)	Odds ratio of change between 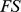 with and without splice junction library
GSNAP	98.94	97.88	98.27	96.19	97.53	1.59
BLAT	96.07	98.20	98.24	92.01	93.01	1.16
BFAST	97.07	99.73	93.30	94.85	96.69	1.59
Bowtie2	99.58	92.60	93.76	90.75	95.06	1.96
SHRiMP2	99.98	93.08	99.99	91.76	99.56	20.38
Novoalign	100.00	81.37	100.0	82.65	99.48	40.07
BWA	100.00	25.11	34.06	33.67	48.04	1.82
BWAMEM	99.92	97.17	98.24	93.18	97.50	2.86
BWASW	99.92	91.97	93.92	89.78	94.74	2.05
STAR	99.99	85.63	94.31	86.92	96.59	4.26

Summary of alignment results with and without a splice junction library for each aligner, in terms of their precision, sensitivity, and the 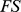 score for reads simulated from the human reference sequence (chr1) using RiSER€s simulation framework. All reads were aligned to the chr1 reference using parameter value settings described in section 5 of the Materials S1.

#### Simulation of reads mapping to the human host genomic regions not represented in the hg19 reference genome

In order to evaluate the impact of reads that originate from human host genomic regions not represented in the human hg19 reference genome and that could align to viral reference sequences (i.e. part 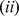 of our benchmark dataset), we simulated 61076 reads (100 bp in length) from chrN_random and chrUn_ chromosomes accessed from the UCSC website (http://hgdownload.cse.ucsc.edu/goldenPath/hg19/chromosomes/). For each aligner, we first aligned all 61076 reads to the human hg19 reference sequences, of those reads that did not align, we determined the number of reads that do align to any of the 4195 viral reference genomes. As shown in Table 9 the number of false positives (i.e host human reads not represented in the human hg19 reference genome that align to any of the viral reference sequences) is the highest for BLAT followed by BFAST and GSNAP. More importantly results presented in Table 9 indicate that the impact that those reads could have on viral characterization is relatively small for most aligners, except for BLAT.

**Table 9:** Human reads simulated from host genomic regions not represented in the human (hg19) reference genome.

Aligner	Number of reads not mapping to hg19 (aligners' moderate sensitivity parameter values)	Number of reads mapping to viral genomes (aligners' default parameter values)	Number of reads mapping to viral genomes (aligners' moderate sensitivity parameter values)	Number of reads mapping to viral genomes (aligners' high sensitivity parameter values)
GSNAP	26759	2	12	12
BLAT	44266	2035	2035	2211
BFAST	1331	113	113	113
Bowtie2	7943	0	2	2
SHRiMP2	4491	0	0	0
Novoalign	10376	37	0	0
BWA	48584	0	0	0
BWAMEM	7621	0	0	0
BWASW	14635	1	1	2
STAR	15088	0	0	1

Summary of alignment results for 61076 reads simulated from host genomic regions not represented in the human (hg19) reference genome. The columns 2–5 indicate the number of reads mapping to 4195 viral genomes and not mapping to the hg19 reference genome (see the first column). The 61076 reads simulated from host genomic regions not represented in the human (hg19) were aligned to the hg19 reference genome as described in section 5 of the Materials S1.

### Runtime measurements

Table 10 shows the time needed for each aligner to align 20 million reads simulated from the human reference genome (hg19) to 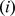 the human (hg19) genome and cDNA reference sequences (downloaded from the Ensembl database) and 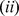 4195 viral reference sequences (see Materials and Methods section). The fastest runtimes were achieved by BWA-MEM, Bowtie2, BWA, STAR and Novoalign.

**Table 10:** Runtime measurements.

Aligner	Human reference runtime (hrs)	Max mem used (GB)	Number of AMD 64 bit core processors	Viral reference runtime (hrs)	Max mem used (GB)	Number of AMD 64 core processors
BWAMEM	0.29	13	17	0.83	3	7
Bowtie2	0.62	9	17	0.06	2	7
BWA	0.66	9	17	0.03	2	7
STAR	0.79	40	17	1.0	5	7
Novoalign	2.1	13	17	0.9	2	7
BWASW	2.68	12	17	0.65	3	7
GSNAP	9.1	12	17	1.3	3	7
BLAST	9.4	12	17	4.4	2	7
SHRiMP2	10.5	46	17	0.4	4	7
BFAST	18.0	32	17	3.0	4	7
BLAT	74.0	5	1	3.0	4	1

The elapsed (wallclock) time needed to align 20 million Illumina reads from a human transcriptome sample against a human (hg19) genome and cDNA reference sequences and 4195 viral reference sequences, sorted according to the first column. For the detailed description of parameter value settings used for each aligner see sections 3 in the Materials S1.

### Evaluation of aligners using real RNA-Seq datasets

In this section we assess the ability of aligners to detect viral transcripts in two real RNA-Seq datasets and compare it to results obtained using our simulated dataset with moderate sensitivity parameter value settings. For the first dataset, we prepared an RNA-Seq library by isolating RNA from human pancreatic duct epithelial (HPDE) cells that were immortalized by transfection with the E6/E7 gene of human papilloma virus 16 [33]. The isolated RNA was sequenced using an Illumina Genome Analyzer IIx to generate 80 million single end reads of 100 bp in length. The RNA-Seq read set was then aligned against 4195 different viral reference sequences using each of the eight aligners (with the exception of BLAST, because of its impractical runtime). In Table 11 we show the alignment results obtained for each aligner (using moderate sensitivity parameter value settings as shown in sections 4 of the Materials S1) across the entire HPV16 reference sequence (RefSeq: NC_001526.2) and across the E6/E7 gene regions using 80 million reads sequenced from the transfected human pancreatic duct epithelial cells.

**Table 11:** Alignment results across the HPV16 and the E6/E7 gene regions.

Aligner	Total number of reads aligned across the HPV16 genome	HPV16 genome coverage (%)	Total coverage across HPV16 E6/E7 gene regions (%)	Average depth of coverage across HPV16 E6/E7 gene regions
GSNAP	52518	33.64	100.00	3730
BFAST	53481	17.31	98.58	5230
BLAT	17068	15.50	94.57	1186
Bowtie2	53521	11.46	93.80	3720
SHRiMP2	9548	9.98	93.02	751
Novoalign	123	8.81	84.76	10
BWA	1	1.2	1	0.12
BWAMEM	17456	10.23	93.80	1226
BWASW	17077	9.83	93.80	1207
STAR	918	9.92	91.47	83

The alignment results obtained for each aligner across the HPV16 reference genome (RefSeq:NC_001526.2) including the E6 and E7 gene regions using 80 million reads sequenced from the transfected human pancreatic duct epithelial cells. All reads were aligned to 4195 viral reference genomes using aligners' moderate sensitivity parameter values (see section 4 in the Materials S1).

**Figure 5:**
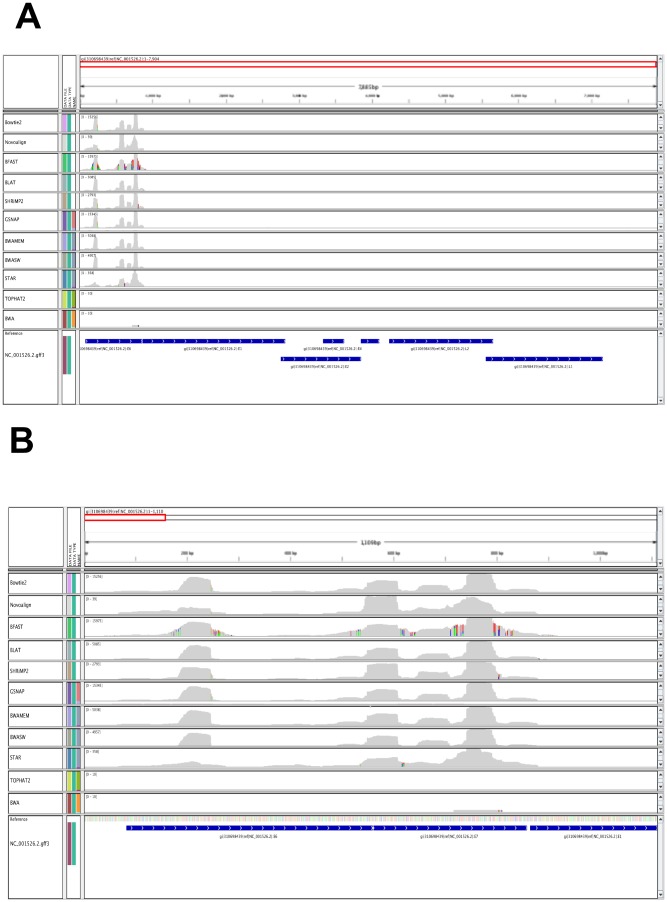
Coverage plot of the HPV16 E6/E7 gene regions. The coverage plot of HPV16 E6/E7 gene regions (NC_001526.2) for each aligner using RNA-Seq data sequenced from the transfected (HPV16-E6/E7) human pancreatic duct epithelial cells.

Ranking aligners according to the coverage and the number of reads aligned across the HPV16 genome we find that GSNAP and BFAST perform best. Six other aligners show similar coverage (BLAT, Bowtie2, BWA-SW, BWA-MEM, SHRiMP2 and STAR) though with different number of reads aligned. Surprisingly Novoalign shows a marked difference in the number of reads aligned (but not coverage) from the rest of aligners (excluding BWA) while BWA aligns a single read across the HPV16 reference sequence. In Figure 5 we show a genome browser view of data presented in Table 11. From Figure 5 we infer that the E6/E7 gene regions expressed in the transfected HPDE cell line have a high degree of sequence similarity to the E6/E7 gene regions of the NC_001526.2 reference sequence. These results, obtained using a real dataset, show a good agreement with results obtained from our simulated study where GSNAP and BWA were ranked respectively second highest (STAR being the highest) and lowest, when used for characterizing viral sequences with low mutation rates (see Table 4 with mutation rate 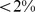). The only important deviation from the ranking presented in Table 4 is found for Novoalign which aligns a significantly smaller number of reads (see Table 11) than expected (see Table 3).

Our second dataset is composed of two RNA-Seq files (106 million reads in total) containing reads sequenced from the HCC cancer obtained from a patient with serologic evidence of HBV infection and available from The Cancer Genome Atlas (TCGA) database [34]. In Table 12 we show that all aligners (using moderate sensitivity parameter value settings as shown in sections 4 of the Materials S1) achieve a 100% coverage across HBV gene regions. The top five aligners with the highest number of reads aligned across the HBV (RefSeq:NC_003977.1) reference sequence in decreasing order are GSNAP, BFAST, STAR and BLAT. These results are also consistent with our simulated results presented in Table 4 and in Table S11 in Materials S1 for mutation rate 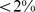 where the best performing algorithms are STAR, GSNAP and BLAT.

**Table 12:** Alignment results across the HBV and its gene regions.

Aligner	Total number of reads aligned across the HBV genome	HBV genome coverage (%)	Total coverage across HBV gene regions (%)	Average depth of coverage across HBV gene regions
GSNAP	53935	99.84	100	1430
BFAST	50163	99.97	100	1372
BLAT	47888	99.32	100	1369
Bowtie2	45195	99.34	100	1270
SHRiMP2	38297	100	100	1036
Novoalign	36273	100	100	1017
BWA	43454	99.72	100	1297
BWAMEM	45043	97.92	100	1282
BWASW	42632	98.41	100	1205
STAR	49019	100	100	1357

The alignment results obtained for each aligner across the HBV reference genome (RefSeq:NC_003977.1) and its gene regions using 106 million reads sequenced from the HCC cancer obtained from a patient with serologic evidence of HBV infection and available from The Cancer Genome Atlas (TCGA). All reads were aligned to 4195 viral reference genomes using aligners' moderate sensitivity parameter values (see section 4 in the Materials S1).

## Discussion

We developed RiSER, a realistic RNA-Seq simulation and evaluation framework, to evaluate eleven aligners (BLAST, BLAT, BWA, BWA-SW, BWA-MEM, BFAST, SHRiMP2, Bowtie2, GSNAP, Novoaligna and STAR) for characterizing viral sequences in transcriptome samples. We proposed and used a new combined measure 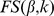, to rank the overall performance of aligners in the context of viral sequence characterization and discovery. We showed that results generated with RiSER are consistent with those obtained using real RNA-Seq datasets in terms of the ranking, with the exception of Novoalign which shows an unexpected drop in the number of reads aligned in real datasets. Because RiSER can also simulate reads across known viral splice junctions, our analysis indicates that splicing in viruses will not significantly impact their characterization when using the alignment approach behind digital subtraction. Even if the assumption of uniform distribution of expression levels of viral transcripts in our model is very approximate (viruses are known to express different transcripts in different amounts [28], [29]) our model suggests that the ranking of aligners remains virtually unchanged as 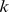 varies between 0 (no reads crossing splice junction regions) and 1 (all reads crossing splice junctions), except for Novoalign which scores significantly lower for mutation rates 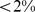 when 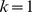 (data shown in Tables S6, S9 and S15 in Materials S1).

In addition to RNA splicing, we evaluated the extent to which aligners can be used for characterizing mutated viral sequences using three distinct parameter value settings for each aligner (default (D), moderate sensitivity (MS) and high sensitivity (HS)). Using our combined 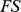 measure we found that BLAST (HS parameter value settings), BFAST (MS parameter value settings), SHRiMP2 (MS parameter value settings), BWA-SW (HS parameter value settings), GSNAP (HS parameter value settings) and BLAT (HS parameter value settings) achieved the best overall performances in characterizing viral sequences at higher mutation rates (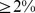). We find BLAST to be the best aligner for characterizing viral genomes in terms of the 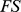 score, sensitivity and overall genome coverage. BLAST however might be impractical to run (i.e., long runtime with very large number of reads) unless parallelized. Furthermore BLAST is not designed to align NGS short reads against reference genomes using base qualities, which could significantly increase the alignment error [32]. BFAST is the second most sensitive aligner of all the aligners tested and can be used effectively in the search and discovery of highly mutated viral sequences with up to 20% (overall) mutation rate. However, BFAST shows a drop in precision as the mutation rate decreases, with 71% precision at the 0% mutation rate (see Table S7 in Materials S1). In principle, this drop in precision can be corrected by ranking genomes based on their coverage, and by considering only those with the highest coverage. On the other hand, for viral genomes with high mutation rates, SHRiMP2, BWA-SW, GSNAP and BLAT achieve high precision (for SHRiMP2 (see Table S7 in Materials S1), for BWA-SW, GSNAP and BLAT see Table S13 in Materials S1) at the expense of lower coverage (for SHRiMP2 see Table 5, for BWA-SW, GSNAP and BLAT see Table S12 in Materials S1), and our results indicate that for these four aligners the best performance in terms of the 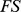 score and coverage will be obtained for viral genomes in the 0%–15% mutation range.

We used our results to identify the best set of aligners (among the eleven considered in this study) for characterizing viruses using digital subtraction. To do this we evaluated the ability of individual aligners to subtract human sequences from the human host using RiSER by simulating a realistic human transcriptome dataset containing 2 million short reads. Using this dataset we applied the RiSER's evaluation framework to ten aligners (BLAT, BWA, BWA-SW, BWA-MEM, BFAST, SHRiMP2, Bowtie2, GSNAP, Novoalign and STAR), since BLAST is not the best suitable aligner for aligning a large number of short NGS reads against a single human reference genome[12], [32].

When information about splice junctions was not available, our results showed that GSNAP has the best performance and scores highest according to the 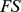 measure (see Table 8) when aligning RNA-Seq human reads back to the human reference and across splice junctions, followed by BFAST and BWA-MEM. If we compare results from Table 7 to results obtained with a different RNA-Seq simulator (BEER [19]) designed for benchmarking aligners using simulated human RNA-Seq data, we find good agreement in the ranking of aligners in terms of the base level accuracy and the number of reads aligned across splice junctions. To compare results between these two studies we first transformed data from Table 7 to calculate the base level accuracy using all of our aligned reads (i.e., reads crossing splice junction and those that do not). Among aligners that are common to both studies (i.e., GSNAP, BFAST, BLAT, BWA and Novoalign) we find that GSNAP achieves the highest base level accuracy followed in the decreasing order by BFAST, BLAT, Novoalign and BWA. This is the same ranking reported in the BEER paper [19], although the base level accuracy values reported for each aligner in the BEER paper are slightly higher than in our study. We also found that GSNAP had the highest number of reads aligned across splice junction regions followed by BLAT (see Table 7) which is also in agreement with results reported in the BEER paper. Together, these results lend support to the validity of our simulation approach.

If the human splice junction library is available then the two aligners that benefit the most from this information (in terms of the number of human reads subtracted, alignment accuracy and 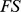 score) are Novoalign and SHRiMP2.

If we assume that the great majority of reads from a human transcriptome sample have a high sequence similarity to the human reference sequence, then our results indicate that:

- if the splice junction library is not available, GSNAP (see Tables 7–8) gives the best overall performance (in terms of the high proportion of reads aligned, high alignment accuracy, ability to align reads across splice junctions and high 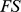 score)
- if the splice junction library with sequences overlapping exon-exon boundaries is available, Novoalign and SHRiMP2 (though with longer runtime) (see Tables 7–8) show the best performance (in terms of the high proportion of reads aligned, 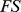 score, high alignment accuracy and fastest runtime)

The candidate non-human sequences that are left can then be aligned against the database of viral reference sequences using, in decreasing order of sensitivity and 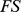 score, BLAST (HS parameter value settings), BFAST (MS parameter value settings), SHRiMP2 (MS parameter value settings), BWA-SW (HS parameter value settings), GSNAP (HS parameter value settings) and BLAT (HS parameter value settings). More importantly, the results in Tables 4 and 5 suggest that combining two algorithms in succession should improve the overall sensitivity and precision of alignments results obtained with any single aligner. We thus suggest a two step approach for aligning candidate non-human sequences. Candidate non-human sequences should first be aligned using a more conservative and faster aligner (such as BWA-MEM, Bowtie2 or Novoalign), the remaining un-aligned reads can then be aligned with a more tolerant aligner such as BLAST, BFAST or SHRiMP2. We also evaluate the potential impact that sequences originating from human host genomic regions, but not represented in the human hg19 reference genome, could have on viral detection (in terms of potential false positive) and found the effect to be small for most of the aligners.

Our results indicate that a clear advantage for detecting mutated viral sequences is conferred by the ability of algorithms to perform local alignments. In our study these included BLAST, BLAT, BFAST, SHRiMP2, GSNAP, BWA-SW, BWA-MEM, Bowtie2 and STAR. BFAST (MS parameter value settings) and SHRiMP2 (MS parameter value settings) show similar results and high sensitivity when compared to other local aligners mentioned above (with the exception of BLAST) due to their ability to align highly polymorphic reads (see Figure 3) with high accuracy (see Figure 4). Though BFAST is more sensitive than SHRiMP2 for more polymorphic reads it is also less accurate. BFAST is also less precise than SHRiMP2 for reads that present fewer mismatches as shown in Tables S7 and S8 in Materials S1. As shown in Figure 4 of all the above local aligners Bowtie2 has the steepest drop in sensitivity as a function of the mutation rate. This result can be explained by the restricted number of mismatches that Bowtie2 allows in a seed alignment during multiseed alignment, while the same restriction is not present in the other local alignment tools mentioned above. Similar results to Bowtie2 were obtained with the recently developed BWA-MEM algorithm though with slightly higher sensitivity and lower accuracy (see Figures 3 and 4) for more polymorphic reads. We also found BWA-MEM to be less sensitive and slightly less accurate (see Tables 3 and 4) than the BWA-SW algorithm.

As expected local aligners that use a Burrows-Wheeler Transform (BWA, BWA-SW, BWA-MEM and Bowtie2) are at least an order of magnitude faster than the rest of the aligners, with the exception of the STAR algorithm which uses sequential maximum mappable seed search in uncompressed suffix arrays, but also has a much larger memory footprint (see Table 10).

Finally, our results suggest that methods that rely on a particular choice of alignment algorithms for viral characterization (such as RINS or PATHSEQ) will be limited in their performance by the alignment algorithm used, and that this choice needs to be made based on a quantitative evaluation method that systematically compares alignment tools in the appropriate context. We believe that future efforts to detect viral sequences in transcriptome samples will benefit from this study's guidance on the choice of the aligner algorithm used. We also hope that our simulation and evaluation framework will be used in the benchmarking of other alignment tools. We note that novel viruses with no sequence similarity to any known reference sequences will be missed with the approach used in our study. In connection with this, a useful complementary approach may be to use a 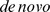 assembly of unaligned reads to generate longer contigs that could be more easily characterized than individual short reads [7]. We also believe that it will be useful to compare results for pathogen detection using NGS technologies to those obtained with the DNA microarrays [35] since they occupy a middle ground between narrowly focused assays such as multiplex PCR and much more broad high-throughput sequencing approaches such as those presented in this study.

## Materials and Methods

The RiSER simulation and evaluation framework is implemented in Python, and is composed of two main parts, an RNA-Seq simulation that wraps around the ART simulator to produce the “true” alignment dataset and the evaluation part used to evaluate the performance of each individual aligner by comparing their alignment results to the true alignment dataset (RiSER can be accessed from github at https://github.com/oicr-ibc/riser).

We chose the eleven alignment algorithms based on the following criteria:

- Basic local alignment tools such as BLAST (used by PATHSEQ) and BLAT (used by RINS) for viral sequence characterization.
- A range of popular short read aligners that are representative of the two main alignment techniques used, namely hash tables (Novoalign, SHRiMP2, GSNAP and BFAST), and suffix arrays, compressed (using Burrows-Wheeler Transform (BWT), including BWT extended FM-indices (BWA, BWA-SW, BWA-MEM and Bowtie2)), and uncompressed (STAR).
- Capacity of the aligner to perform gapped alignments (true for all of the above aligners).
- Capacity of the aligner to align reads across splice junctions (GSNAP, BLAT, BLAST and BFAST and STAR).

In the following sections we describe how RNA-Seq data is simulated from mutated and non-mutated viral reference sequences and the human reference genome, and the evaluation approach we use to assess aligners' performance.

### Simulation

For each known viral genome the RNA-Seq simulation starts by extracting the reference sequence and sequence information such as ‘gene’ or ‘CDS’ from the FEATURES field contained in the GenBank flat file [36] (see Figure S1 in Materials S1). Alternatively, as in the case of the human reference genome, RiSER can also process any custom-provided reference sequence (in FASTA format) and a transcript information file. This information is then used to generate two files, one containing individual DNA transcript sequences in FASTA format and the second containing information about junctions, including junction number, junction coordinates and individual transcripts' start and stop coordinates. The junction information file is used to determine the accuracy with which aligners align reads across known splice junctions.

The transcript sequence file is then fed to the ART [18] simulator (parameters: art_illumina [Illumina platform] -l [read length = 100 bp] -f [the fold of read coverage to be simulated = 10] -sam [generate SAM alignment file], with default values for indels and substitution rates) which generates single-end (or pair-end) reads and base quality values. For the Illumina type of sequencing ART simulates realistic reads where base quality score is position dependent (the mean quality score decreases as function of increasing base position) and where base substitution is simulated according to the empirical position-dependent distribution of base quality score, measured in large training datasets. The base quality score does not directly provide information for INDEL errors, and ART simulates insertion and deletion errors directly from empirical distributions based on training data [18].

As output ART generates a file with cDNA reads in the FASTQ format and an alignment file in the SAM format. The alignment file generated by ART serves as the true alignment to which the output of all the other aligners is compared (see Figure S1 in Materials S1).

In order to test aligners' performances as a function of viral mutation rates our simulation approach also enables generation of reads from mutated viral genomes at different rates. Each viral reference sequence is first mutated using a random substitution model at the pre-specified rate; the mutated reference sequence is then used to generate transcript sequences using ART in the same way as a non-mutated viral reference sequence (see Figure S1 in Materials S1).

### Evaluation

To evaluate the performance of different aligners the FASTQ files generated by RiSER's RNA-Seq simulation (see the previous section) were first aligned against a (non-spliced) viral reference genome (or human reference genome) and the aligner's output file was then compared to the true alignment file generated by ART (see Figure S2 in Materials S1).

#### Definition of the alignment accuracy and other metrics

In order to determine the accuracy with which an aligner aligns a simulated read to a reference genome, we require that each base position of the read aligns to the right location (obtained from the true alignment file), and that the CIGAR (for more information about the CIGAR format see [37]) operation (i.e., one of the I, D, N, S, H, P,  =  and X) given by the aligner for that base is identical to the one in the true alignment file (note that for the BLAST algorithm we convert its output alignment format to the SAM format in order to obtain the corresponding CIGAR codes). The per-read alignment accuracy A is thus measured and expressed as the percentage of correctly aligned bases out of the total number of bases contained by the read. The alignment accuracy of any given set of reads is defined as the average alignment accuracy (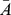) of that read set.

When reads were being aligned to a single viral genome (such as shown in Figure 1) and in the case of multiple read alignments we chose the alignment with the best alignment score and counted it as a success. In the case where alignment scores were indistinguishable (i.e., a read aligning to multiple locations with the same alignment scores on a single viral genome) one alignment was chosen at random and counted as a success.

As noted above, for reads that cross a splice junction we used the information from the splice junction information file (see Figure S2 in Materials S1) to determine the accuracy of alignment across that junction. We also allowed a single read to span more than one splice junction.

Each aligner was run with three different sets of parameter value settings; default, moderate sensitivity and high sensitivity (detailed command lines used for all aligners, version numbers and all parameters are shown in the Materials S1) except for Novoalign which was run with default parameters only and BFAST run with moderate sensitivity and high sensitivity parameter value settings since for BFAST the default and moderate sensitivity parameter value settings are identical. The reasons for using only default parameter values for Novoalign are two fold: first, Novoalign adjusts some of its parameter values based on the input (for example the genome size is used for determining the k-mer length necessary for indexing the genome) and second, there is no simple way of changing the number of mismatches permitted without changing the values for the gap open and gap extended penalty which we were reluctant to do with respect to the parameter settings used by other aligners.

The metrics we used to assess the performance of each aligner were as follows:

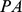
 (percentage of aligned reads not crossing splice junctions) = 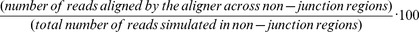

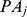
 (percentage of aligned reads crossing splice junctions) = 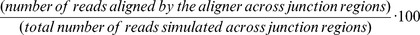

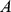
 (percentage alignment accuracy per read) = 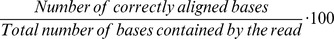

4. Average alignment accuracy 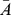 for reads aligned outside splice junction regions
5. Average alignment accuracy 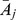 for reads aligned across splice junctions

In addition to the five metrics stated above, we calculated precision (i.e., 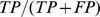) which measured the aligner's ability to align reads to the right reference sequence and sensitivity (or recall) (i.e., 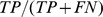) which measured the aligner's ability to align reads to the right reference sequences weighted by the total number of reads that fail to align. TP (true positives), FP (false positives) and FN (false negatives) are defined as follows:

- TP = number of reads aligning to the right reference sequence
- FP = number of reads aligning to a different reference sequence from the one they were derived from
- FN = number of reads that do not align to any reference sequence

To estimate the FP rate we aligned simulated reads derived from each of the four viral sequences shown in Table 1 and the human reference genome (see Table 6) to a complete set of 4195 known viral reference sequences downloaded from NCBI. Note that in this case all aligners were run by allowing multiple alignments for each read to be reported. In this case if any of these alignments is the correct alignment we counted it as a success (or a TP). Low precision (i.e., high false positive rate) indicates aligner's propensity to align short reads to other reference sequences in addition to the one they were generated from, making the detection of the target genome more difficult due to an increase in noise (i.e., in the number of additional falsely reported reference sequences). Low sensitivity (i.e., high false negative rate) indicates that an aligner fails to align a significant number of reads back to the reference sequence they were generated from.

To assess the variability of our simulated data and our assessment metrics we repeated each simulation (for each given viral genome) ten times, producing ten different FASTQ files. We note that in our simulation framework we did not attempt to accurately quantify and simulate expression levels of viral transcripts (which for most viruses are not known) or human transcripts, instead we generated reads with uniform coverage across transcripts and junctions, in order to test the mapping ability of each aligner. However even with our simpler approach we expected to find more reads crossing splice junctions for viruses that exhibit splicing, than those that do not.

## Supporting Information

Materials S1
**Supporting text, figures and tables. Figure S1. Shows the diagram of steps used for RNA-Seq data simulation in the RiSER's framework. Figure S2. Shows RiSER's evaluation procedure (for each aligner) and metrics used (see** **Materials and Methods** **section) in the RiSER's framework.** **Figure S3. Histogram plot of the number of aligned reads as a function of the viral mutation rate (default parameter value settings).** Histogram plot of the number of aligned reads obtained using default parameter value settings as a function of the mutation rate for each aligner averaged over the four viral genomes (see Table 1). **Figure S4. Histogram plot of the number of aligned reads as a function of the viral mutation rate (high sensitivity parameter value settings).** Histogram plot of the number of aligned reads obtained using high sensitivity parameter value settings as a function of the mutation rate for each aligner averaged over the four viral genomes (see Table 1). **Figure S5. Histogram plot of the average accuracy as a function of the viral mutation rate (default parameter value settings).** Histogram plot of the average accuracy obtained using default parameter value settings as a function of the mutation rate for each aligner averaged over the four viral genomes (see Table 1.). **Figure S6. Histogram plot of the average accuracy as a function of the viral mutation rate (high sensitivity parameter value settings).** Histogram plot of the average accuracy obtained using high sensitivity parameter value settings as a function of the mutation rate for each aligner averaged over the four viral genomes (see Table 1.). **Table S1. Summary of S1 and S2 scores for viral reference sequences (default parameter value settings).** Shows the summary of alignment results aggregated over four viral genomes for non-mutated viral reference sequences and sorted by S1 score. The average alignment accuracy for reads crossing splice junctions (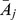) and those not crossing splice junctions (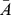) is defined in the Materials and Methods section. **Table S2. Summary of values for the** 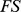 **measure (default parameter value settings).** Shows values of the 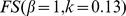 measure averaged over four viral genomes (see Table 2) as a function of viral mutation rates, sorted according to the average 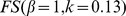 values for mutation rates 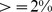. **Table S3. Genome Coverage (default parameter value settings).** The summary of values of genomes' coverage in terms of their minimum and maximum values (i.e., min-max) obtained by each individual aligner, averaged over four viral genomes (see Table 2) as a function of viral mutation rates and sorted according to the average coverage for mutation rates 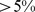. **Table S4. Precision (default parameter value settings).** Shows values for precision (see Materials and Methods section for the definition of precision) for each aligner as a function of the viral sequence mutation rate. **Table S5. Sensitivity (default parameter value settings).** Shows values for sensitivity (or recall) (see Materials and Methods section for the definition of sensitivity) for each aligner as a function of the viral sequence mutation rate. **Table S6. FS measure for** 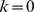 **and** 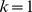 **(default parameter value settings).** Shows the change in values of the 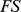 measure for each individual aligner when aligning reads generated from viral sequences without (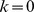) and with splicing (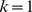) (see also Table 2 and Table S1 in Materials S1). **Table S7. Precision (moderate sensitivity parameter value settings).** Shows values for precision (see Materials and Methods section for the definition of precision) for each aligner as a function of the viral sequence mutation rate. **Table S8. Sensitivity (moderate sensitivity parameter value settings).** Shows values for sensitivity (or recall) (see Materials and Methods section for the definition of sensitivity) for each aligner as a function of the viral sequence mutation rate. **Table S9 FS measure for** 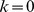 **and** 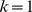 **(moderate sensitivity parameter value settings).** Shows the change in values of the 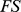 measure for each individual aligner when aligning reads generated from viral sequences without (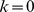) and with splicing (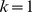) (see also Tables 2 and 3). **Table S10. Summary of S1 and S2 scores for viral reference sequences (high sensitivity parameter value settings).** Shows the summary of alignment results aggregated over four viral genomes for non-mutated viral reference sequences and sorted by S1 score. The average alignment accuracy for reads crossing splice junctions (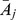) and those not crossing splice junctions (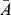) is defined in the Materials and Methods section. **Table S11. Summary of values for the** 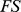 **measure (high sensitivity parameter value settings).** Shows values of the 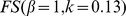 measure averaged over four viral genomes (see Table 2) as a function of viral mutation rates, sorted according to the average 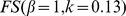 values for mutation rates 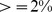. **Table S12. Genome Coverage (high sensitivity parameter value settings).** The summary of values of genomes' coverage in terms of their minimum and maximum values (i.e., min-max) obtained by each individual aligner, averaged over four viral genomes (see Table 2) as a function of viral mutation rates and sorted according to the average coverage for mutation rates 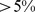. **Table S13. Precision (high sensitivity parameter value settings).** Shows values for precision (see Materials and Methods section for the definition of precision) for each aligner as a function of the viral sequence mutation rate. **Table S14. Sensitivity (high sensitivity parameter value settings).** Shows values for sensitivity (or recall) (see Materials and Methods section for the definition of sensitivity) for each aligner as a function of the viral sequence mutation rate. **Table S15 FS measure for** 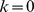 **and** 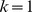 **(high sensitivity parameter value settings).** Shows the change in values of the 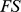 measure for each individual aligner when aligning reads generated from viral sequences without (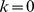) and with splicing (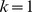) (see also Table 2 and Table S10).(PDF)
