# Supplementary material for: Evaluation of Alignment Algorithms for Discovery and Identification of Pathogens Using RNA-Seq
Source: PLoS One. 2013 Oct 30;8(10):e76935. doi: 10.1371/journal.pone.0076935 (PMC3813700; doi:10.1371/journal.pone.0076935)
Supplement: Materials S1 — Supporting text, figures and tables. Figure S1. Shows the diagram of steps used for RNA-Seq data simulation in the RiSER's framework. Figure S2. Shows RiSER's evaluation procedure (for each aligner) and metrics used (see Materials and Methods section) in the RiSER's framework. Figure S3. Histogram plot of the number of aligned reads as a function of the viral mutation rate (default parameter value settings). Histogram plot of the number of aligned reads obtained using default parameter value settings as a function of the mutation rate for each aligner averaged over the four viral genomes (see Table 1). Figure S4. Histogram plot of the number of aligned reads as a function of the viral mutation rate (high sensitivity parameter value settings). Histogram plot of the number of aligned reads obtained using high sensitivity parameter value settings as a function of the mutation rate for each aligner averaged over the four viral genomes (see Table 1). Figure S5. Histogram plot of the average accuracy as a function of the viral mutation rate (default parameter value settings). Histogram plot of the average accuracy obtained using default parameter value settings as a function of the mutation rate for each aligner averaged over the four viral genomes (see Table 1.). Figure S6. Histogram plot of the average accuracy as a function of the viral mutation rate (high sensitivity parameter value settings). Histogram plot of the average accuracy obtained using high sensitivity parameter value settings as a function of the mutation rate for each aligner averaged over the four viral genomes (see Table 1.). Table S1. Summary of S1 and S2 scores for viral reference sequences (default parameter value settings). Shows the summary of alignment results aggregated over four viral genomes for non-mutated viral reference sequences and sorted by S1 score. The average alignment accuracy for reads crossing splice junctions () and those not crossing splice junctions () is defined in the Materials [file pone.0076935.s001.pdf]

Supplemental Materials S1 - Evaluation of alignment algorithms for discovery and identification of pathogens using RNA-Seq.

Ivan Borozan, Stuart Watt, Vincent Ferretti.

September 24, 2013

Contents

1 RiSER framework **1**

2 Version of aligners used **1**

3 Parameter value settings used for runtime measurements **2**

4 Parameter value settings for aligners (reads simulated from viral genomes) **5**

5 Parameter value settings for aligners (reads simulated from the chr1 (hg19) refer

6 Supplementary Tables **10**
- 6.1 Results obtained with aligners' default parameter value settings: 10
- 6.2 Results obtained with aligners' moderate sensitivity parameter value settings: 14
- 6.3 Results obtained with aligners' high sensitivity parameter value settings: 16

7 Supplementary Figures **21**

1 RiSER framework

The RISER framework is available as open source on the github's webpage https://github.com/oicr-ibc/riser which also includes a full set of instructions for installing and running RiSER. The github's wiki page for this project contains links to all the datasets used in the manuscript and can be accessed here https://github.com/oicr-ibc/riser/wiki.

2 Version of aligners used

Novoalign (2.07.14)
BFAST (0.7.0a)
BWA (0.7.4-r385)
SHRiMP2 (2.2.2)
Bowtie2 (2.1.0)
GSNAP (2012-07-03)
BLAT (v.34)
BLAST (2.2.25+)
STAR (2.3.0)

3 Parameter value settings used for runtime measurements

BFAST
indexing:
First ten indexes for each genome(s) were created using the following commands:

bfast fasta2brg -f genome.fa;
I=1;
for MASK in 1111111111111111111111 1111101110111010100101011011111
10111110101101001011000011010001111111 10111001101001100100111101010001011111
11111011011101111011111111 111111100101001000101111101110111 1111010111001010001010111[illegible]
111101101011011001100000101101001011101 1111011010001000110101100101100110100111
1111010010110110101110010110111011
do
bfast index -f genome.fa -i $I -w 14 -m $MASK -n 4;
let I=I+1;
done

Viral:
bfast match -f genome.fa -r single_end_reads.fq > bfast_match_out
bfast localalign -U -f genome.fa -m bfast_match_out > bfast_localalign_out.baf
bfast postprocess -a 4 -f genome.fa -i bfast_localalign_out.baf
Human(hg19+cDNA):
bfast match -f genome.fa -r single_end_reads.fq > bfast_match_out
bfast localalign -U -f genome.fa -m bfast_match_out > bfast_localalign_out.baf
bfast postprocess -a 4 -f genome.fa -i bfast_localalign_out.baf

BLAT
Viral:
blat -t=dna -q=rna -minScore=20 -stepSize=5 -maxIntron=750000 -makeOoc=11.ooc genome.fa single_end_reads.fasta output.psl
blat -t=dna -q=rna -tileSize=11 -minIdentity=90 -minScore=20 -stepSize=5 -maxIntron=750000 -ooc=11.ooc
Human(hg19+cDNA):
blat -t=dna -q=rna -minScore=20 -stepSize=5 -maxIntron=750000 -makeOoc=11.ooc genome.fa single_end_reads.fasta output.psl
blat -t=dna -q=rna -tileSize=11 -minIdentity=90 -minScore=20 -stepSize=5 -maxIntron=750000 -ooc=11.ooc

BLAST
Viral:
blastall -a 7 -p blastn -d genome.fa -m 8 -i single_end_reads.fasta -o output_BLAST.txt
Human(hg19+cDNA):
blastall -a 17 -p blastn -d genome.fa -m 8 -i single_end_reads.fasta -o output_BLAST.txt

Bowtie2
indexing:
bowtie2-build genome.fa genome

Viral:
bowtie2 -p 7 –local -k 100 -x genome -U single_end_reads.fq -S output.sam
Human(hg19+cDNA):
bowtie2 -p 17 –local -x genome -U single_end_reads.fq -S output.sam

BWA
indexing:
bwa index -a bwtsw genome.fa

Viral:
bwa aln -t 7 genome.fa single_end_reads.fq > forward.sai
bwa samse genome.fa forward.sai single_end_reads.fq
Human(hg19+cDNA):
bwa aln -t 17 genome.fa single_end_reads.fq > forward.sai
bwa samse genome.fa forward.sai single_end_reads.fq

BWAMEM
indexing:
same as for BWA

Viral:
bwa mem -t 7 -r 1.0 genome.fa single_end_reads.fq
Human(hg19+cDNA)
bwa mem -t 17 -r 1.0 genome.fa single_end_reads.fq

BWASW
indexing:
same as for BWA

Viral:
bwa bwasw -t 7 -z 5 genome.fa single_end_reads.fq
Human(hg19+cDNA):
bwa bwasw -t 17 -z 5 genome.fa single_end_reads.fq

GSNAP
indexing:
gmap_build -d genome genome.fa

Viral:
cat single_end_reads.fq | gsnap -m 10 -i 2 -N 1 -t 7 -A sam -D Genome_directory -d genome
Human(hg19):
cat single_end_reads.fq | gsnap -m 10 -i 2 -N 1 -t 17 -A sam -D Genome_directory -d genome

Novoalign
indexing:
novoindex genome.nix genome.fa

Viral:
novoalign -d genome.nix -f single_end_reads.fq -o SAM
Human(hg19+cDNA):
novoalign -d genome.nix -f single_end_reads.fq -o SAM

SHiMP2
indexing:
genome_file_name=genome.fa

Viral:
gmapper-ls –local -N 7 –qv-offset 33 single_end_reads.fq genome_file_name
Human(hg19+cDNA):
gmapper-ls –local -N 17 –qv-offset 33 single_end_reads.fq genome_file_name

STAR
indexing:
Viral:
star –runMode genomeGenerate –genomeDir /genome/star GenomeDir –genomeFastaFiles genome.fa –genomeChrBinNbits 18 –runThreadN 8
Human(hg19+cDNA):

star –runMode genomeGenerate –genomeDir /genome/star GenomeDir –genomeFastaFiles genome.fa –genomeChrBinNbits 14 –runThreadN 8

Viral:
star –genomeDir /genome/star –readFilesIn single_end_reads.fq –winAnchorMultimapNmax 200 –outSAMunmapped Within –alignIntronMax 20000 –seedSearchStartLmax 25 –outFilterMultimapNmax 100
Human(hg19+cDNA):
star –runThreadN 17 –genomeDir /genome/star –readFilesIn single_end_reads.fq

4 Parameter value settings for aligners (reads simulated from viral genomes)

BFAST

Default/Moderate Sensitivity:
bfast match -K 8 -M 384 -f genome.fa -r single_end_reads.fq > bfast_match_out
bfast localalign -M 384 -U -f genome.fa -m bfast_match_out > bfast_localalign_out.baf
bfast postprocess -a 4 -f genome.fa -i bfast_localalign_out.baf
Reporting multiple alignments
bfast postprocess -a 1 -f genome.fa -i bfast_localalign_out.baf

High Sensitivity:
bfast match -K 8 -M 1280 -f genome.fa -r single_end_reads.fq > bfast_match_out
bfast localalign -M 1280 -U -f genome.fa -m bfast_match_out > bfast_localalign_out.baf
bfast postprocess -a 4 -f genome.fa -i bfast_localalign_out.baf
Reporting multiple alignments
bfast postprocess -a 1 -f genome.fa -i bfast_localalign_out.baf

BLAT
Default:
blat -t=dna -q=rna -minScore=30 -maxIntron=750000 -makeOoc=11.ooc genome.fa single_end_reads.fasta output.psl
blat -t=dna -q=rna -tileSize=11 -minIdentity=90 -minScore=30 -stepSize=11 -maxIntron=750000 -ooc=11.ooc

Moderate sensitivity:
blat -t=dna -q=rna -minScore=30 -stepSize=5 -maxIntron=750000 -makeOoc=11.ooc

genome.fa single_end_reads.fasta output.psl
blat -t=dna -q=rna -tileSize=11 -minIdentity=90 -minScore=30 -stepSize=5 -maxIntron=750000 -ooc=11.ooc

High sensitivity:
blat -t=dna -q=rna -minIdentity=85 -minScore=20 -stepSize=5 -maxIntron=750000 -makeOoc=11.ooc genome.fa single_end_reads.fasta output.psl
blat -t=dna -q=rna -tileSize=11 -minIdentity=85 -minScore=20 -stepSize=5 -maxIntron=750000 -ooc=11.ooc

BLAST
Low sensitivity:
blastn -word_size 16 -task megablast -query=single_end_reads.fasta -db=genome.fa -evalue 0.0000001

Moderate sensitivity:
blastn -word_size 10 -task megablast -query=single_end_reads.fasta -db=genome.fa -evalue 0.0000001

High sensitivity:
blastn -word_size 7 -task megablast -query=single_end_reads.fasta -db=genome.fa -evalue 0.0000001

Bowtie2

Default:
bowtie2 -k 100 -x genome -U single_end_reads.fq -S output.sam

Moderate sensitivity:
bowtie2 –local -k 100 -x genome -U single_end_reads.fq -S output.sam

High sensitivity:
bowtie2 –very-sensitive-local -N 1 -k 100 -x genome -U single_end_reads.fq -S output.sam

BWA
indexing:
bwa index -a is genome.fa

Default:
bwa aln genome.fa single_end_reads.fq > forward.sai

bwa samse genome.fa forward.sai single_end_reads.fq > output.sam

Moderate sensitivity:
bwa aln aln -n 5 -e 10 -o 3 -l 22 genome.fa single_end_reads.fq > forward.sai
bwa samse genome.fa forward.sai single_end_reads.fq > output.sam

High sensitivity:
bwa aln -n 7 -e 10 -o 3 -l 1000 genome.fa single_end_reads.fq > forward.sai
bwa samse genome.fa forward.sai single_end_reads.fq > output.sam

BWAMEM

Default:
bwa mem genome.fa single_end_reads.fq > output.sam
Reporting multiple alignments
bwa mem -a genome.fa single_end_reads.fq > output.sam

Moderate sensitivity:
bwa mem -r 1.0 genome.fa single_end_reads.fq > output.sam
Reporting multiple alignments
bwa mem -a -r 1.0 genome.fa single_end_reads.fq ¿ output.sam

High sensitivity:
bwa mem -r 0.5 genome.fa single_end_reads.fq > output.sam
Reporting multiple alignments
bwa mem -a -r 0.5 genome.fa single_end_reads.fq > output.sam

BWASW
indexing:
same as for BWA

Default:
bwa bwasw genome.fa single_end_reads.fq > output.sam

Moderate sensitivity:
bwa bwasw -z 5 genome.fa single_end_reads.fq > output.sam

High sensitivity:
bwa bwasw -z 10 genome.fa single_end_reads.fq > output.sam

GSNAP

Default:
cat single_end_reads.fq | gsnap -A sam -D Genome_directory -d genome

Moderate sensitivity:
cat single_end_reads.fq | gsnap -m 10 -i 2 -N 1 -A sam -D Genome_directory -d genome

High sensitivity:
cat single_end_reads.fq | gsnap -m 12 -i 2 -N 1 -A sam -D Genome_directory -d genome

Novoalign

Default:
novoalign -d genome.nix -f single_end_reads.fq -o SAM
Reporting multiple alignments
novoalign -d genome.nix -f single_end_reads.fq -o SAM -r All

SHiMP2

Default:
gmapper-ls –qv-offset 33 single_end_reads.fq genome_file_name

Moderate sensitivity:
gmapper-ls –qv-offset 33 –local single_end_reads.fq genome_file_name

High sensitivity:
gmapper-ls -V -w 150% -n 1 -r 50% -l 40% -Z -h 60% -a -1 –qv-offset 33 –local single_end_reads.fq genome_file_name

STAR
indexing:
STAR –runMode genomeGenerate –genomeSAindexNbases 2 –genomeDir /genome/star GenomeDir –genomeFastaFiles genome.fa

Default:
star –genomeDir /genome/star –readFilesIn single_end_reads.fq –winAnchorMultimapNmax 200 –outSAMunmapped Within –alignIntronMax 20000 –outFilterMultimapNmax 100

Moderate sensitivity:
star –genomeDir /genome/star –readFilesIn single_end_reads.fq –winAnchorMultimapNmax 200 –outSAMunmapped Within –alignIntronMax 20000 –seedSearchStartLmax 25 –outFilterMultimapNmax 100

High sensitivity:
star –genomeDir /genome/star –readFilesIn single_end_reads.fq –winAnchorMultimapNmax 200 –outSAMunmapped Within –alignIntronMax 20000 –seedSearchStartLmax 12 –outFilterMultimapNmax 100 –outFilterMismatchNmax 20

5 Parameter value settings for aligners (reads simulated from the chr1 (hg19) reference)

BFAST
bfast match -K 8 -M 384 -f genome.fa -r single_end_reads.fq > bfast_match_out
bfast localalign -M 384 -U -f genome.fa -m bfast_match_out > bfast_localalign_out.baf
bfast postprocess -a 4 -f genome.fa -i bfast_localalign_out.baf

BLAT
blat -t=dna -q=rna -minScore=20 -stepSize=5 -maxIntron=750000 -makeOoc=11.ooc genome.fa single_end_reads.fasta output.psl
blat -t=dna -q=rna -tileSize=11 -minIdentity=90 -minScore=30 -stepSize=5 -maxIntron=750000 -ooc=11.ooc

Bowtie2
bowtie2 –local -x genome -U single_end_reads.fq -S output.sam

BWA
bwa aln aln -n 5 -e 10 -o 3 -l 22 genome.fa single_end_reads.fq > forward.sai

BWAMEM
bwa mem -r 1.0 genome.fa single_end_reads.fq > output.sam

BWASW
bwa bwasw -z 5 genome.fa single_end_reads.fq > output.sam

GSNAP
cat single_end_reads.fq | gsnap -m 10 -i 2 -N 1 -A sam -D Genome_directory -d genome

Novoalign
novoalign -d genome.nix -f single_end_reads.fq -o SAM

SHiMP2
gmapper-ls –qv-offset 33 –local single_end_reads.fq genome_file_name

STAR
star –genomeDir /genome/star –readFilesIn single_end_reads.fq –seedSearchStartLmax 25

6 Supplementary Tables

6.1 Results obtained with aligners' default parameter value settings:

Summary of S1 and S2 scores for viral reference sequences

Aligner	PA (percentage of aligned reads (non-junctions)) (%)	PA_j (percentage of aligned reads (junctions)) (%)	$\bar{A}$ non-junctions (%)	$\bar{A_j}$ (junctions) (%)	S1	S2
Novoalign	99.96	21.62	99.92	92.52	99.94	44.73
SHRiMP2	99.97	56.74	99.89	76.15	99.93	65.73
BFAST	99.94	99.97	98.82	70.16	99.38	83.75
BLAST	98.43	99.38	97.41	70.28	97.92	83.57
BWASW	98.25	95.26	95.98	70.24	97.11	81.8
BWAMEM	96.83	94.24	96.7	69.2	96.77	80.76
STAR	94.57	98.36	98.79	97.5	96.65	97.93
BLAT	94.57	91.8	95.12	77.51	94.84	84.35
Bowtie2	84.98	29.17	100	85.39	92.18	49.91
BWA	33.89	1.75	99.96	96.68	58.21	13.01
GSNAP	33.94	69.75	95.05	64.99	56.8	67.33

Table S1: Shows the summary of alignment results aggregated over four viral genomes for non-mutated viral reference sequences and sorted by S1 score. The average alignment accuracy for reads crossing splice junctions ($\bar{A_j}$) and those not crossing splice junctions ($\bar{A}$) is defined in the Materials and Methods section.

Summary of values for the FS measure

Aligner	Mutation (0%)	Mutation (2%)	Mutation (5%)	Mutation (10%)	Mutation (15%)	Mutation (20%)	Mutation (25%)
BFAST	89.87	90.14	91	87.79	73.78	50.53	25.64
SHRiMP2	94.72	94.43	93.76	86.76	63.29	26.27	5.86
BLAST	92.86	92.36	89.89	76.52	56.35	35.9	13.73
BWAMEM	96.06	92.48	83.69	56.48	27.6	8.49	1.73
BWASW	94.73	90.44	80.73	53.2	26.16	7.8	1.49
STAR	98.05	95.08	83.07	44.81	13.19	0.96	0
BLAT	95.75	89.53	73.44	39.9	15.4	5.63	1
Novoalign	96.27	95.19	83.85	33.17	3.7	0.08	0
Bowtie2	88.62	79.21	60.64	29.52	7.08	0.65	0
GSNAP	54.77	35.95	17.86	9.95	7.99	4.66	1.47
BWA	51.78	28.15	6.69	0.38	0	0	0

Table S2: Shows values of the $FS(\beta = 1, k = 0.13)$ measure averaged over four viral genomes (see Table.2) as a function of viral mutation rates, sorted according to the average $FS(\beta = 1, k = 0.13)$ values for mutation rates >= 2%.

Genome Coverage

Aligner	Mutation (0%)	Mutation (2%)	Mutation (5%)	Mutation (10%)	Mutation (15%)	Mutation (20%)	Mutation (25%)
BFAST	97-100	97-100	96-100	97-100	94-98	76-85	49-55
BLAST	97-100	97-100	96-100	93-99	78-87	52-67	24-28
SHRiMP2	97-100	97-100	96-100	97-100	86-93	43-54	6-12
BWASW	97-100	97-100	96-100	94-96	60-67	20-25	0-8
BWAMEM	97-100	97-100	94-100	78-83	31-42	7-11	0-3
Bowtie2	97-100	96-100	94-99	53-82	15-49	1-21	0-4
STAR	97-100	97-100	96-100	71-74	21-28	0-4	0-0
BLAT	97-100	96-100	83-99	59-68	19-22	4-6	0-1
Novoalign	97-100	97-100	92-99	45-54	0-10	0-1	0-0
GSNAP	96-97	66-76	24-39	9-18	5-10	4-6	0-2
BWA	96-97	63-69	7-19	0-1	0-0	0-0	0-0

Table S3: The summary of values of genomes' coverage in terms of their minimum and maximum values (i.e., min-max) obtained by each individual aligner, averaged over four viral genomes (see Table.2) as a function of viral mutation rates and sorted according to the average coverage for mutation rates > 5%.

Precision

Aligner	Mutation (0%)	Mutation (2%)	Mutation (5%)	Mutation (10%)	Mutation (15%)	Mutation (20%)	Mutation (25%)
BLAST	83.12	85.90	89.54	92.42	93.76	96.01	97.98
BFAST	70.94	73.38	79.34	86.05	86.97	90.07	86.6
BLAT	99.64	99.25	99.2	98.95	96.97	98.95	73.3
Bowtie2	97.09	97.28	98.31	98.73	99.27	75	0
BWA	99.68	99.65	99.18	50	0	0	0
GSNAP	96.18	91.74	90.22	93.48	88.9	98.13	71.87
Novoalign	99.68	99.53	99.71	99.37	75	25	0
SHRiMP2	88.54	89.95	92.61	96.06	97.82	98.29	98.98
BWASW	99.83	99.72	99.81	99.55	99.46	99.74	74.09
BWAMEM	96.67	96.43	97.11	97.83	97.67	98.47	73.19
STAR	99.66	99.5	99.57	99.44	99.57	49.69	0

Table S4: Shows values for precision (see Materials and Methods section for the definition of precision) for each aligner as a function of the viral sequence mutation rate.

Sensitivity

Aligner	Mutation (0%)	Mutation (2%)	Mutation (5%)	Mutation (10%)	Mutation (15%)	Mutation (20%)	Mutation (25%)
BLAST	97.56	95.68	90.38	66.68	39.92	19.46	5.32
BFAST	99.86	99.87	98.92	90.41	65.28	34.05	13.06
BLAT	96.55	86.94	62.24	24.94	6.72	1.7	0.25
Bowtie2	84.82	69.34	44.11	14.62	2.2	0.1	0
BWA	34.43	13.67	1.86	0.05	0	0	0
GSNAP	35.24	18.3	7.29	3.67	2.94	1.47	0.32
Novoalign	100	99.68	80.19	17.60	0.99	0.01	0
SHRiMP2	99.99	99.92	99.13	86.87	48.94	12.91	1.89
BWASW	89.39	78.58	59.14	25.87	8.46	1.53	0.27
BWAMEM	98.21	92.57	78.14	41.14	14.6	2.9	0.62
STAR	98.92	94.53	74.22	26.7	4.51	0.12	0

Table S5: Shows values for sensitivity (or recall) (see Materials and Methods section for the definition of sensitivity) for each aligner as a function of the viral sequence mutation rate.

FS measure for $k = 0$ and $k = 1$

Aligner	$FS(\beta = 1, k = 0)$	$FS(\beta = 1, k = 1)$	Odds ratios of change between $k = 1$ and $k = 0$
BLAST	93.75	86.61	2.32
BFAST	90.8	83.35	1.97
BLAT	96.44	90.95	2.7
Bowtie2	91.36	67.22	5.15
BWA	54.58	25.8	3.46
GSNAP	54.12	58.93	0.82
Novoalign	99.89	66.82	448.97
SHRiMP2	96.88	78.57	8.47
BWASW	95.7	87.84	3.08
BWAMEM	97.1	88.7	4.26
STAR	97.96	98.61	0.68

Table S6: Shows the change in values of the FS measure for each individual aligner when aligning reads generated from viral sequences without ($k = 0$) and with splicing ($k = 1$) (see also Table.2 and supplementary Table.S1).

6.2 Results obtained with aligners' moderate sensitivity parameter value settings:

Precision

Aligner	Mutation (0%)	Mutation (2%)	Mutation (5%)	Mutation (10%)	Mutation (15%)	Mutation (20%)	Mutation (25%)
BLAST	72.56	75.63	81.28	88.41	92.34	95.65	97.34
BFAST	70.94	73.38	79.34	86.05	86.97	90.07	86.6
BLAT	99.63	99.26	99.09	98.23	95.69	98.53	98.34
Bowtie2	92.38	93.46	95.03	94.98	94.51	96.50	60.56
BWA	99.86	99.53	99.94	47.71	0	0	0
GSNAP	99.47	98.95	97.76	94.57	90.72	90.64	86.96
Novoalign	99.68	99.53	99.71	99.37	75.00	25.00	0
SHRiMP2	87.68	89.30	92.05	95.61	97.51	98.15	98.96
BWASW	99.89	99.79	99.82	99.62	99.48	99.62	74.03
BWAMEM	96.43	96.18	96.82	97.62	97.37	98.11	73.25
STAR	99.66	99.5	99.57	99.49	99.59	49.7	0

Table S7: Shows values for precision (see Materials and Methods section for the definition of precision) for each aligner as a function of the viral sequence mutation rate.

Sensitivity

Aligner	Mutation (0%)	Mutation (2%)	Mutation (5%)	Mutation (10%)	Mutation (15%)	Mutation (20%)	Mutation (25%)
BLAST	98.58	98.78	98.97	96.69	84.66	52.78	20.87
BFAST	99.86	99.87	98.92	90.41	65.28	34.05	13.06
BLAT	97.97	91.18	71.05	34.73	13.03	3.75	1.23
Bowtie2	93.18	81.74	60.99	27.26	11.23	3.88	0.63
BWA	35.76	14.63	2.01	0.05	0	0	0
GSNAP	98.63	91.41	71.99	37.58	19.89	10.46	4.01
Novoalign	100.00	99.68	80.19	17.60	0.99	0.01	0.00
SHRiMP2	99.99	99.92	99.17	88.28	53.07	15.45	2.46
BWASW	96.41	90.25	77.95	41.37	16.54	3.39	0.63
BWAMEM	98.21	92.56	78.11	41.11	14.58	2.89	0.62
STAR	98.91	94.83	74.51	26.71	4.55	0.12	0

Table S8: Shows values for sensitivity (or recall) (see Materials and Methods section for the definition of sensitivity) for each aligner as a function of the viral sequence mutation rate.

FS measure for $k = 0$ and $k = 1$

Aligner	$FS(\beta = 1, k = 0)$	$FS(\beta = 1, k = 1)$	Odds ratios of change between $k = 1$ and $k = 0$
BLAST	90.59	83.61	1.89
BFAST	90.8	83.35	1.97
BLAT	97.19	94.16	2.14
Bowtie2	94.52	85.89	2.83
BWA	55.68	26.4	3.5
GSNAP	97.99	96.12	1.97
Novoalign	99.89	66.82	448.97
SHRiMP2	96.30	84.41	4.80
BWASW	97.69	90.17	4.62
BWAMEM	97.04	88.65	4.2
STAR	97.75	98.44	0.69

Table S9: Shows the change in values of the FS measure for each individual aligner when aligning reads generated from viral sequences without ($k = 0$) and with splicing ($k = 1$) (see also Tables 2 and 3).

6.3 Results obtained with aligners' high sensitivity parameter value settings:

Summary of S1 and S2 scores for viral reference sequences

Aligner	PA (percentage of aligned reads (non-junctions)) (%)	PA_j (percentage of aligned reads (junctions)) (%)	$\bar{A}$ non-junctions (%)	$\bar{A}_j$ (junctions) (%)	S1	S2
Novoalign	99.96	21.62	99.92	92.52	99.94	44.73
BFAST	99.94	99.97	99.45	63.57	99.69	79.72
SHRiMP2	99.42	56.64	98.78	81.86	99.1	68.09
BLAST	99.49	100	97.4	70.47	98.44	83.94
GSNAP	97.96	98.27	97.02	89.78	97.49	93.93
BWASW	98.61	99.14	95.95	69.63	97.27	83.08
Bowtie2	94.15	91.32	99.48	70.52	96.78	80.25
BWAMEM	96.83	94.24	96.7	69.2	96.77	80.76
BLAT	98.17	98.82	95.01	82.74	96.58	90.42
STAR	90.87	95.3	99.04	97.92	94.87	96.6
BWA	66.82	7.2	99.93	92.28	81.71	25.78

Table S10: Shows the summary of alignment results aggregated over four viral genomes for non-mutated viral reference sequences and sorted by S1 score. The average alignment accuracy for reads crossing splice junctions ($\bar{A}_j$) and those not crossing splice junctions ($\bar{A}$) is defined in the Materials and Methods section.

Summary of values for the FS measure

Aligner	Mutation (0%)	Mutation (2%)	Mutation (5%)	Mutation (10%)	Mutation (15%)	Mutation (20%)	Mutation (25%)
BLAST	89.6	90.23	91.21	91.4	87.5	71.66	42.89
BFAST	89.75	90.14	91	87.79	73.78	50.53	25.64
GSNAP	98.25	96.29	89.49	65.82	41.58	25.79	11.3
BLAT	97.68	96.09	90.43	70.62	43.87	21.09	7.63
BWASW	97.24	95.55	91.25	72.6	43.42	14.64	3.28
SHRiMP2	95.59	95.03	92.06	72.09	36.16	6.27	0.74
STAR	97.39	95.34	88.98	66.01	32.3	5.98	0.64
BWAMEM	96	92.42	83.63	56.44	27.58	8.48	1.73
Bowtie2	93.79	88.09	76.45	48.72	27.58	12.21	3.03
Novoalign	96.27	95.19	83.85	33.17	3.7	0.08	0
BWA	77.65	56.97	25.02	3.91	0.08	0	0

Table S11: Shows values of the $FS(\beta = 1, k = 0.13)$ measure averaged over four viral genomes (see Table.2) as a function of viral mutation rates, sorted according to the average $FS(\beta = 1, k = 0.13)$ values for mutation rates $>=$ 2%.

Genome Coverage

Aligner	Mutation (0%)	Mutation (2%)	Mutation (5%)	Mutation (10%)	Mutation (15%)	Mutation (20%)	Mutation (25%)
BLAST	97-100	97-100	96-100	97-100	95-100	93-97	72-78
BFAST	97-100	97-100	96-100	97-100	94-98	76-85	49-55
BWASW	97-100	97-100	96-100	96-99	72-79	33-43	0-10
GSNAP	97-100	97-100	96-100	89-93	55-70	34-46	10-18
BLAT	97-100	97-100	96-100	85-93	53-63	22-28	2-10
SHRiMP2	97-100	97-100	96-100	91-97	58-64	3-17	0-3
Bowtie2	97-100	96-100	94-99	80-86	45-57	14-22	0-7
STAR	97-100	97-100	96-100	78-94	34-54	3-9	0-2
BWAMEM	97-100	97-100	94-100	78-83	31-42	7-11	0-3
Novoalign	97-100	97-100	92-99	45-54	0-10	0-1	0-0
BWA	97-100	87-97	43-59	6-8	0-0	0-0	0-0

Table S12: The summary of values of genomes' coverage in terms of their minimum and maximum values (i.e., min-max) obtained by each individual aligner, averaged over four viral genomes (see Table.2) as a function of viral mutation rates and sorted according to the average coverage for mutation rates $> 5\%$.

Precision

Aligner	Mutation (0%)	Mutation (2%)	Mutation (5%)	Mutation (10%)	Mutation (15%)	Mutation (20%)	Mutation (25%)
BLAST	71.69	74.91	80.59	88.01	92.25	95.69	97.25
BFAST	70.94	73.38	79.34	86.05	86.97	90.07	86.6
BLAT	99.61	99.22	98.77	95.82	88.06	75.24	50.96
Bowtie2	91.79	93.01	94.65	95.18	94.15	96.19	61.58
BWA	99.91	99.76	99.83	99.78	25	0	0
GSNAP	99.61	99.33	98.9	96.27	93.95	93.21	89.26
Novoalign	99.68	99.53	99.71	99.37	75	25	0
SHRiMP2	92.46	93.76	95.86	97.89	99.14	99.34	49.1
BWASW	99.88	99.85	99.8	99.69	99.33	99.65	74.04
BWAMEM	96.42	96.18	96.82	97.61	97.37	98.11	73.31
STAR	99.64	99.51	99.54	99.54	99.68	99.9	50

Table S13: Shows values for precision (see Materials and Methods section for the definition of precision) for each aligner as a function of the viral sequence mutation rate.

Sensitivity

Aligner	Mutation (0%)	Mutation (2%)	Mutation (5%)	Mutation (10%)	Mutation (15%)	Mutation (20%)	Mutation (25%)
BLAST	98.94	99.04	99.3	98.53	88.56	56.26	22.84
BFAST	99.82	99.87	98.89	90.33	65.24	34.05	13.06
BLAT	99.61	98.69	91.54	63.58	32.09	11.05	3.91
Bowtie2	94.1	83.27	64.07	30.37	13.22	4.23	0.83
BWA	68.01	40.29	11.94	0.99	0.01	0	0
GSNAP	99.37	97.25	86.61	53.22	27.51	14.49	5.37
Novoalign	100	99.68	80.19	17.6	0.99	0.01	0
SHRiMP2	99.97	99.75	94.94	61.15	20.4	1.78	0.14
BWASW	98.26	94.63	84.63	50.29	20.15	3.9	0.75
BWAMEM	98.21	92.56	78.11	41.11	14.58	2.89	0.62
STAR	99.85	99.13	93.76	60.08	19.52	1.86	0.14

Table S14: Shows values for sensitivity (or recall) (see Materials and Methods section for the definition of sensitivity) for each aligner as a function of the viral sequence mutation rate.

FS measure for $k = 0$ and $k = 1$

Aligner	$FS(\beta = 1, k = 0)$	$FS(\beta = 1, k = 1)$	Odds ratios of change between $k = 1$ and $k = 0$
BLAST	90.47	83.54	1.87
BFAST	90.93	81.31	2.3
BLAT	98.08	94.9	2.75
Bowtie2	94.84	86.36	2.9
BWA	81.32	45.68	5.18
GSNAP	98.48	96.67	2.24
Novoalign	99.89	66.82	448.97
SHRiMP2	97.57	80.88	9.51
BWASW	98.16	90.72	5.47
BWAMEM	97.04	88.65	4.19
STAR	97.28	98.16	0.67

Table S15: Shows the change in values of the FS measure for each individual aligner when aligning reads generated from viral sequences without ($k = 0$) and with splicing ($k = 1$) (see also Table.2 and supplementary Table.S10).

7 Supplementary Figures

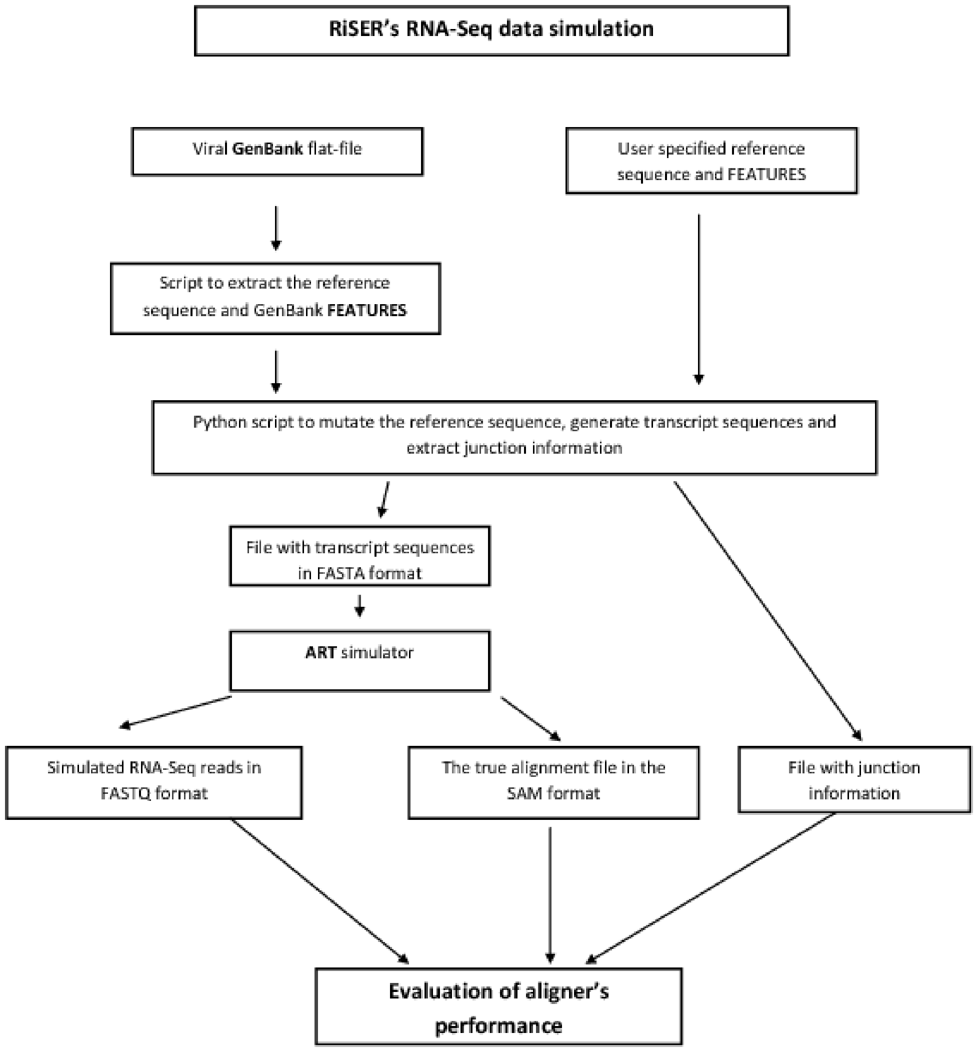

Figure S1: Shows the diagram of steps used for RNA-Seq data simulation in the RiSER's framework.

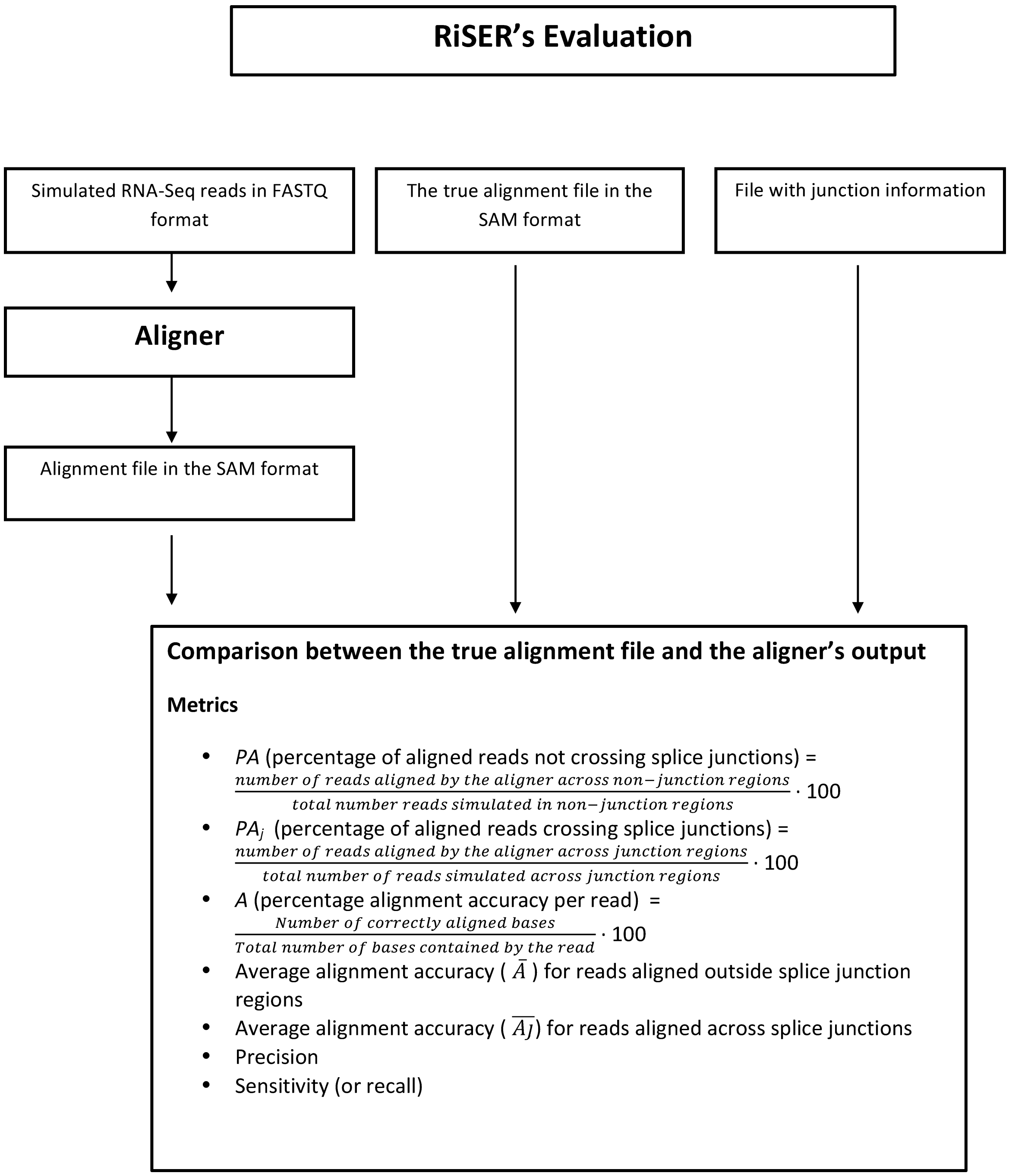

Figure S2: Shows RiSER's evaluation procedure (for each aligner) and metrics used (see Materials and Methods section) in the RiSER framework.

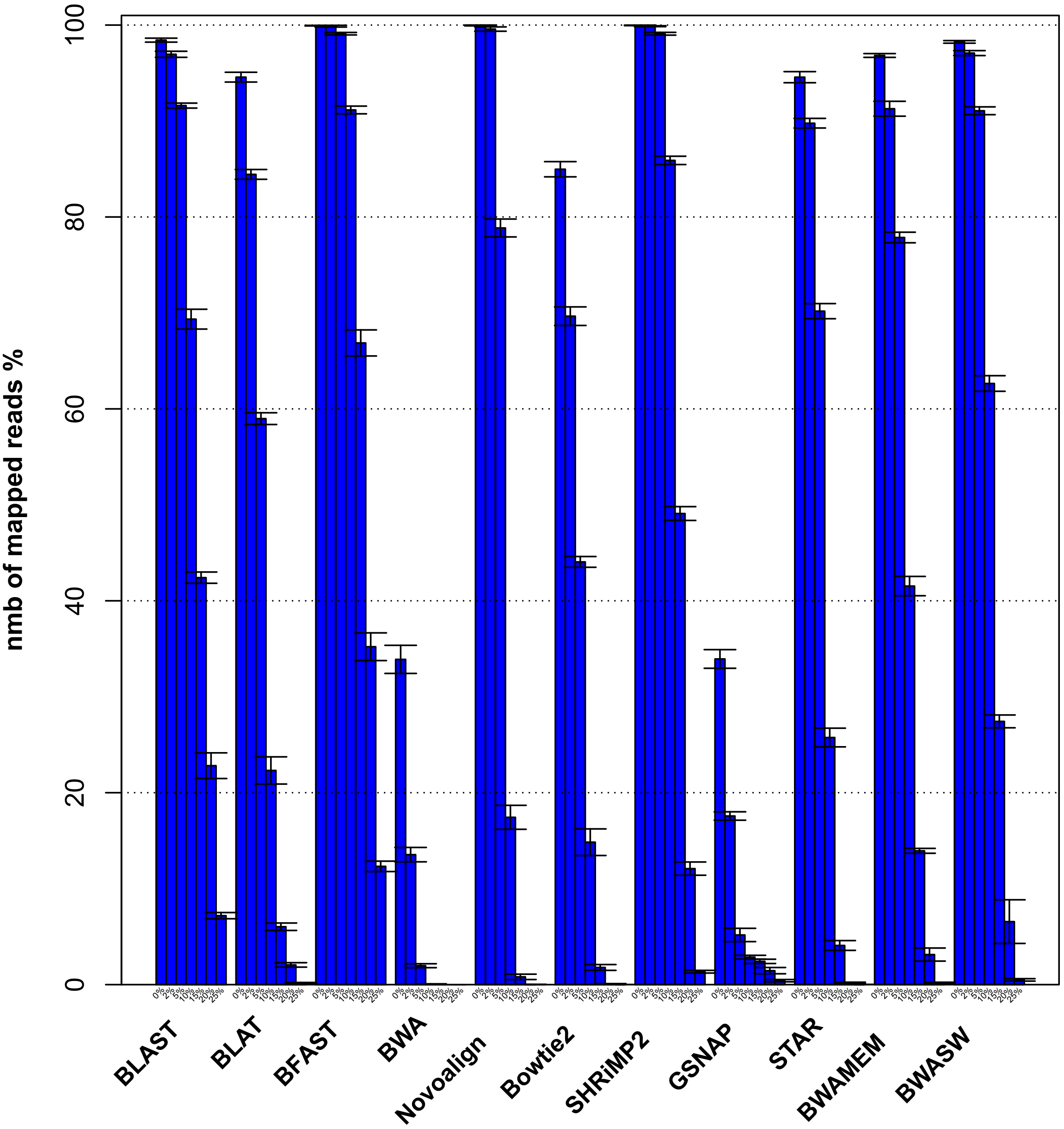

Figure S3: Histogram plot of the number of aligned reads obtained using default parameter value settings as a function of the mutation rate for each aligner averaged over the four viral genomes (see Table.1).

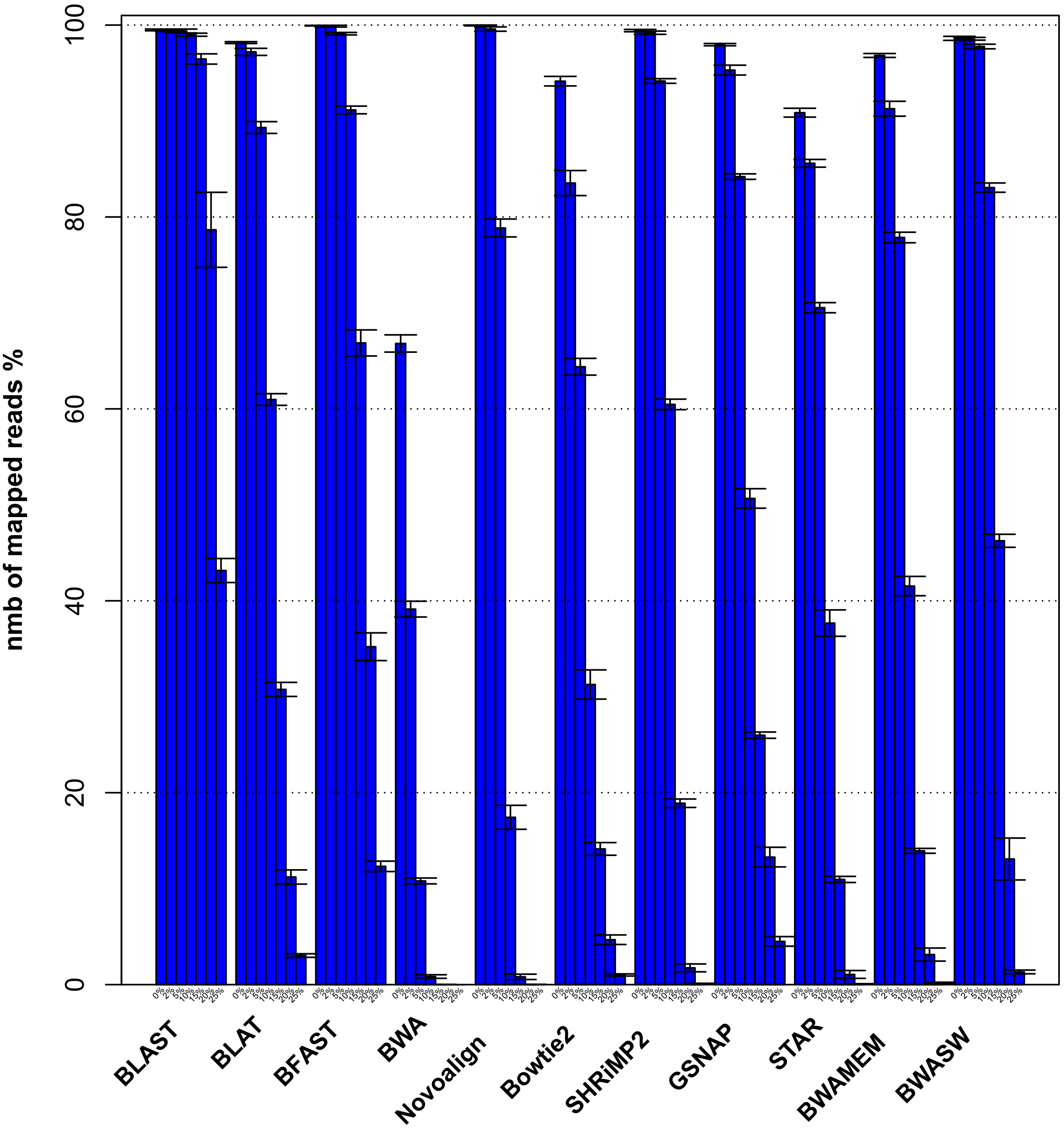

Figure S4: Histogram plot of the number of aligned reads obtained using high sensitivity parameter value settings as a function of the mutation rate for each aligner averaged over the four viral genomes (see Table.1).

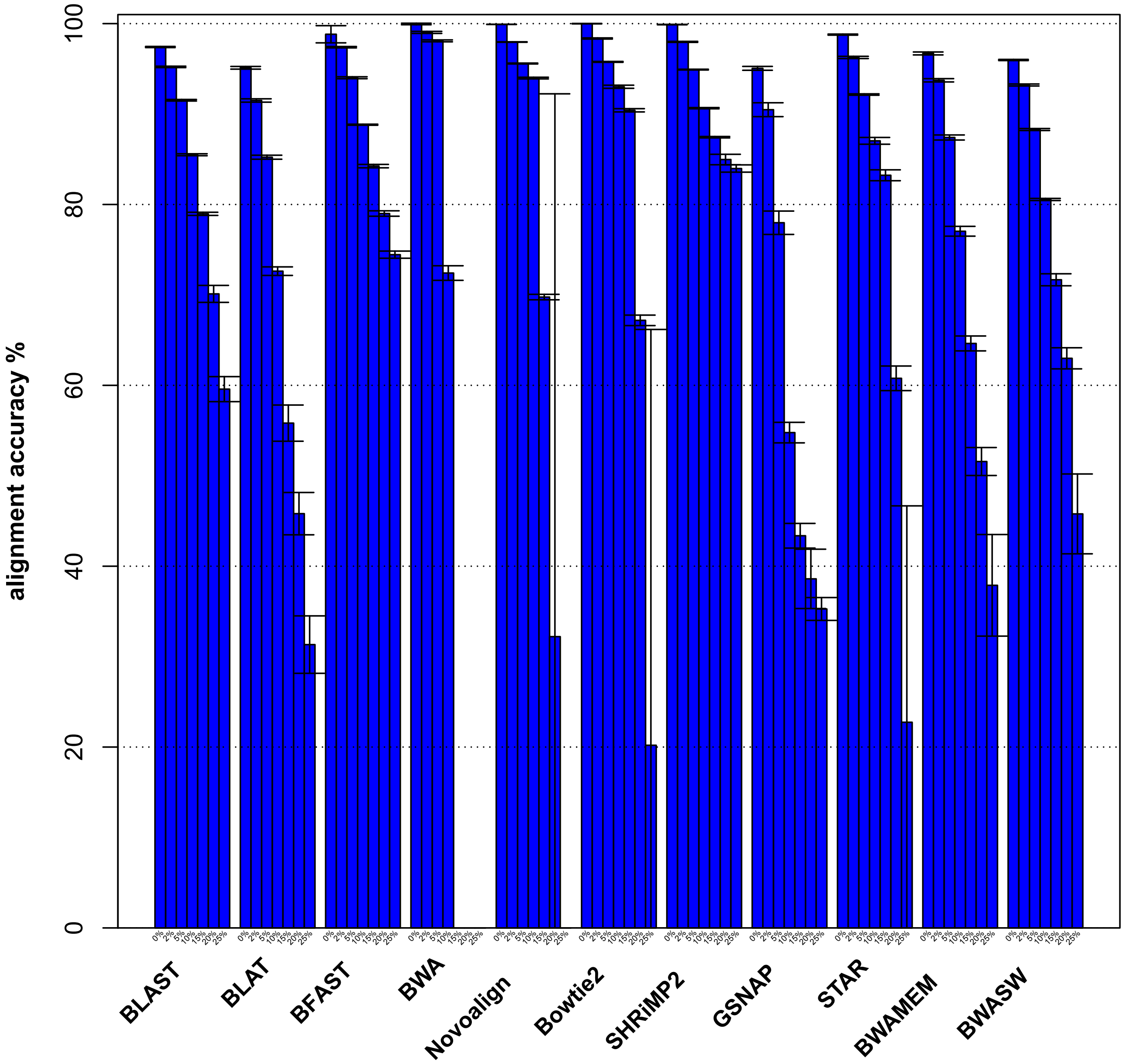

Figure S5: Histogram plot of the average accuracy obtained using default parameter value settings as a function of the mutation rate for each aligner averaged over the four viral genomes (see Table.1.).

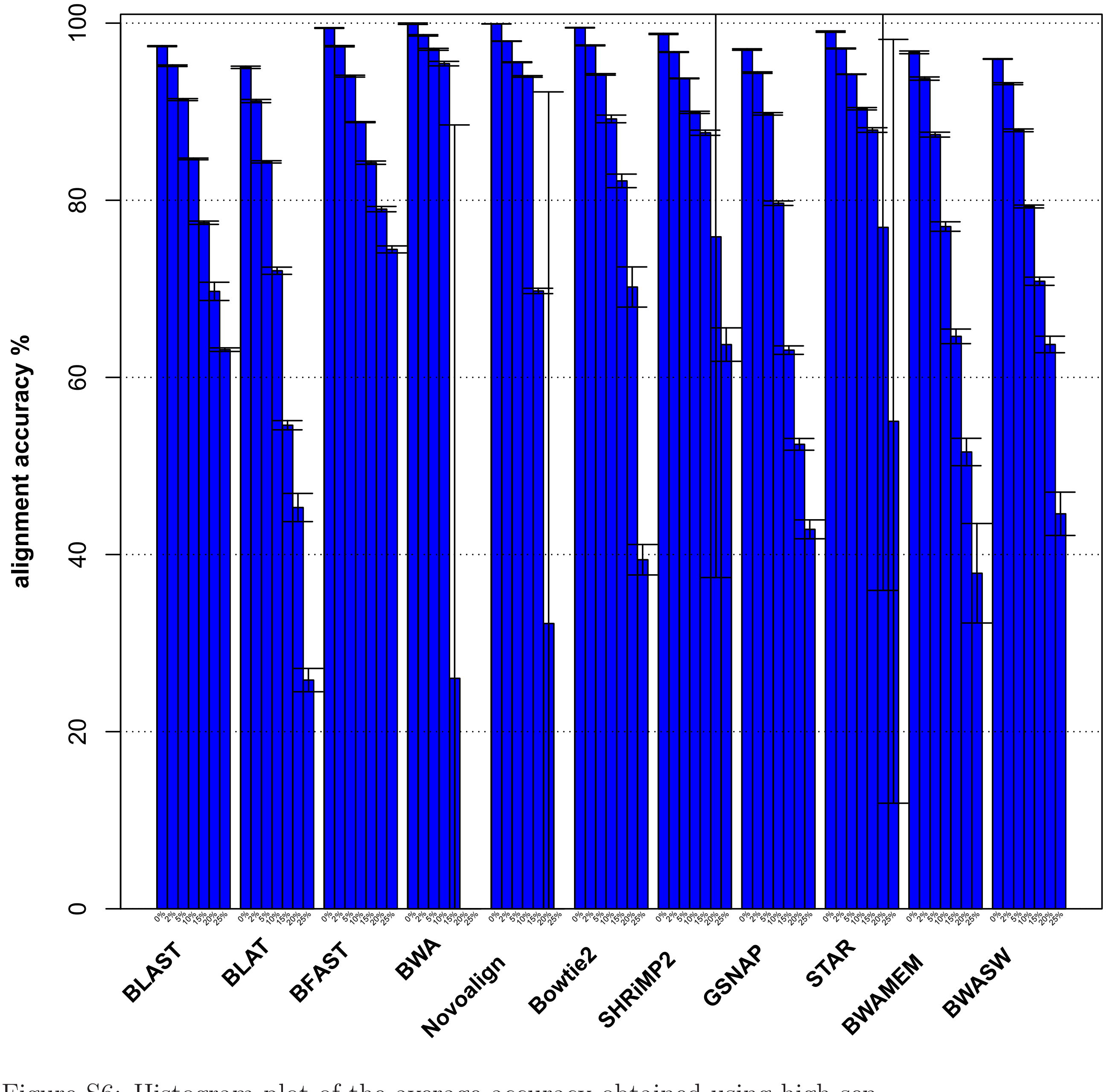

Figure S6: Histogram plot of the average accuracy obtained using high sensitivity parameter value settings as a function of the mutation rate for each aligner averaged over the four viral genomes (see Table.1.).
